# Interdependent roles of PKM2 in photoreceptors and RPE: implications for retinal degeneration

**DOI:** 10.1038/s41419-026-08997-3

**Published:** 2026-06-19

**Authors:** Ammaji Rajala, Rahul Rajala, Larissa J. Trevino, Tyler M. Black, Gennadiy Moiseyev, Michael Kinter, Raju V. S. Rajala

**Affiliations:** 1 Department of Ophthalmology, University of Oklahoma Health Sciences Center, Oklahoma City, OK USA; 2 OU Health Dean McGee Eye Institute, OU Health Campus, Oklahoma City, OK USA; 3 Department of Cell Biology, University of Oklahoma Health Sciences Center, Oklahoma City, OK USA; 4 Oklahoma Medical Research Foundation, Oklahoma City, OK USA; 5 Harold Hamm Diabetes Center, University of Oklahoma Health Sciences Center, Oklahoma City, OK USA; 6 College of Medicine, University of Oklahoma Health Sciences Center, Oklahoma City, OK USA; 7 Department of Biochemistry, Wake Forest University School of Medicine, Winston-Salem, NC USA; 8 Departments of Biochemistry and Physiology, University of Oklahoma Health Sciences Center, Oklahoma City, OK USA

**Keywords:** Molecular neuroscience, Cell death

## Abstract

Pyruvate kinase M2 (PKM2) functions as both a glycolytic enzyme and a transcriptional co-activator that coordinates metabolism and cell survival. Here, we define the developmental timing, cellular distribution, and physiological role of PKM isoforms in the mouse retina. PKM2 expression begins at postnatal day 2, preceding PKM1, and is highly enriched in photoreceptors, whereas PKM1 predominates in retinal ganglion cells. Conditional deletion of PKM2 in the retina, rods, or retinal pigment epithelium (RPE) demonstrated that PKM2 is essential for maintaining retinal structure and function. Loss of PKM2 impaired glycolytic activity, decreased ATP generation, and disrupted metabolic balance, leading to cellular disorganization and degeneration in both photoreceptors and the RPE. In the RPE, PKM2 deficiency decreased RPE65 protein levels and impaired the regeneration of 11-*cis*-retinal, disrupting the visual cycle. PKM2 deletion disrupted the normal cone opsin gradient, indicating that PKM2-dependent metabolic and transcriptional functions are essential for maintaining proper cone organization in the retina. Moreover, rod-specific deletion of PKM2 in Abca4 mutant mice showed early signs of retinal degeneration. The studies described in this manuscript highlight the interdependence of photoreceptor and RPE metabolism and show that PKM2 plays an important role in retinal energy homeostasis and neuronal survival, providing insight into the mechanisms underlying photoreceptor and RPE degeneration in age-related macular degeneration.

**Figure:**
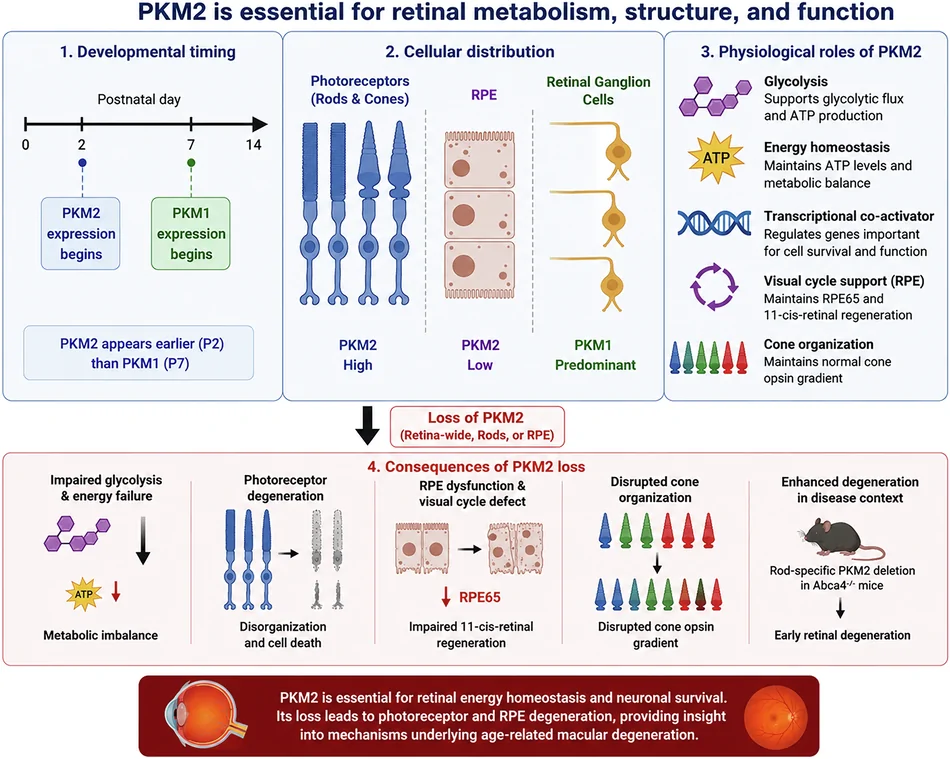

## Introduction

Pyruvate kinase M1 (PKM1) and M2 (PKM2) are alternatively spliced isoforms of the *PKM* gene [1, 2]. These isoforms arise from the mutually exclusive inclusion of exon 9 or exon 10 in the *PKM* transcript [1, 2]. Inclusion of exon 9 produces PKM1, whereas inclusion of exon 10 generates PKM2 [1, 2]. Both PKM1 and PKM2 are enzymes that play roles in glycolysis, but PKM2 also functions as a transcriptional co-activator [3, 4]. In addition, PKM2 is both allosterically and post-translationally regulated, whereas PKM1 is not [5–9]. PKM2 is expressed in various cell types, particularly in those with high metabolic demands, such as mitotic tumor cells, proliferating endothelial cells, and growing fetal tissues [9]. Interestingly, despite differing proliferative states, PKM2 is also highly expressed in non-proliferating postmitotic retinal photoreceptor cells, where it supports aerobic glycolysis to maintain outer segment renewal and anabolic activities, similar to its function in rapidly dividing cells [9–11].

The regulatory flexibility of PKM2 enables it to channel glucose metabolism toward biosynthetic processes and sustain redox balance, vital for both non-dividing and proliferative cells. A metabolic relationship exists between the retinal pigment epithelium (RPE) and photoreceptors, in which glucose transported from the RPE to photoreceptors is converted into lactate [12]. This lactate is then shuttled back to the RPE, where it serves as a fuel source for mitochondrial energy production [12]. In aged mice, age-related macular degeneration (AMD) models, and postmortem RPE from AMD patients, PKM2 expression is higher than in younger RPE [13, 14]. However, the cause of this increase remains unclear. In this study, we examined the developmental expression patterns of PKM1 and PKM2 and analyzed how PKM2 loss affects retinal cell function, structure, and metabolism. We also explored how PKM2 deficiency in the RPE influences RPE integrity and photoreceptor health, and how the absence of PKM2 in photoreceptors alters gene expression and contributes to retinal degeneration in an AMD mouse model.

## Results

### Localization of PKM1 and PKM2 in the developing retina

**Fig. 1:**
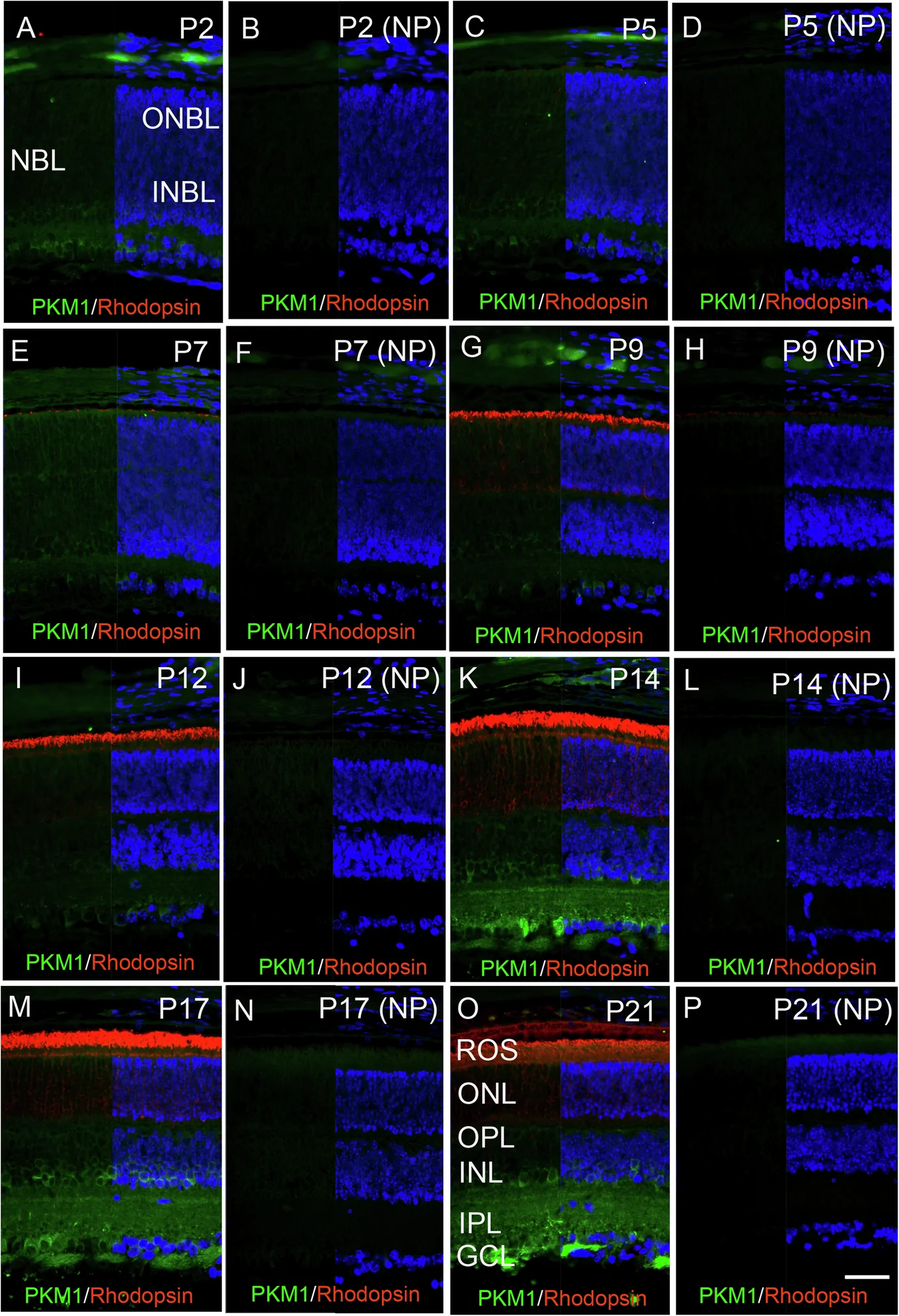
Developmental expression of PKM1 in the retina. Eyes were fixed in Prefer fixative, embedded in paraffin, and sectioned. Retinal sections were collected at postnatal day (P) 2, P5, P7, P9, P12, P14, P17, and P21, and stained with PKM1 (green) and opsin (red) antibodies. **A**, **C**, **E**, **G**, **I**, **K**, **M**, **O** show sections incubated with primary antibodies. **B**, **D**, **F**, **H**, **J**, **L**, **N**, **P** represent no–primary antibody controls. Each panel was divided into two halves, one without DAPI and the other with DAPI. NBL neuroblastic layer, ONBL outer neuroblastic layer, INBL inner neuroblastic layer, ROS rod outer segments, ONL outer nuclear layer, OPL outer plexiform layer, INL inner nuclear layer, IPL inner plexiform layer, GCL ganglion cell layer. Scale bar = 50 μm.

To examine the expression of PKM1 and PKM2 during postnatal development, mouse retinal sections prepared at postnatal days 2 (P2), P7, P9, P12, P14, P17, and P21 were subjected to immunohistochemistry using PKM1 and PKM2 antibodies. The neuroblastic layer (NBL) represents a developmental stage of the retina during which proliferating retinal progenitor cells (neuroblasts) divide and differentiate to generate the various types of retinal neurons. The NBL is further classified into outer NBL (ONBL) and inner NBL (INBL). Weak PKM1 expression was observed at P2 below the INBL (Fig. 1A), which persisted until P7. The expression of the photoreceptor-specific protein rhodopsin begins at P7 (Fig. 1E). During P9 and P12, PKM1 expression shifted to the inner plexiform layer and ganglion cell layer (Fig. 1G, I), where it became strongly expressed at P14 (Fig. 1K). A similar pattern was observed at P17 and P21 in the mature retina (Fig. 1M, O).

**Fig. 2:**
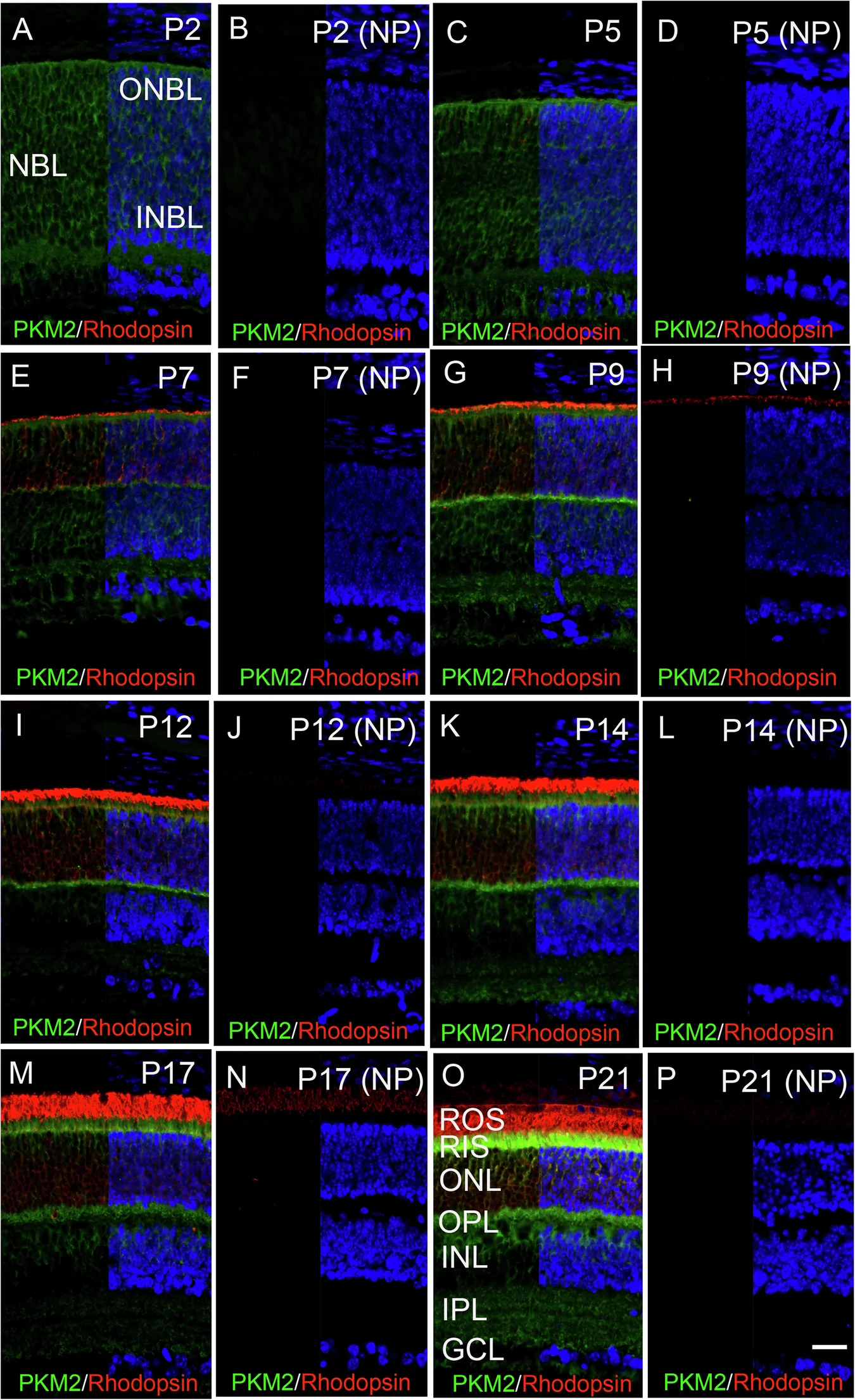
Developmental expression of PKM2 in the retina. Retinal sections from postnatal days P2, P5, P7, P9, P12, P14, P17, and P21 were stained with antibodies against PKM2 (green) and opsin (red). **A**, **C**, **E**, **G**, **I**, **K**, **M**, **O** show sections incubated with primary antibodies. **B**, **D**, **F**, **H**, **J**, **L**, **N**, **P** represent no–primary antibody controls. Each panel was divided into two halves, one without DAPI and the other with DAPI. NBL neuroblastic layer, ONBL outer neuroblastic layer, INBL inner neuroblastic layer. Scale bar = 50 μm.

**Fig. 3:**
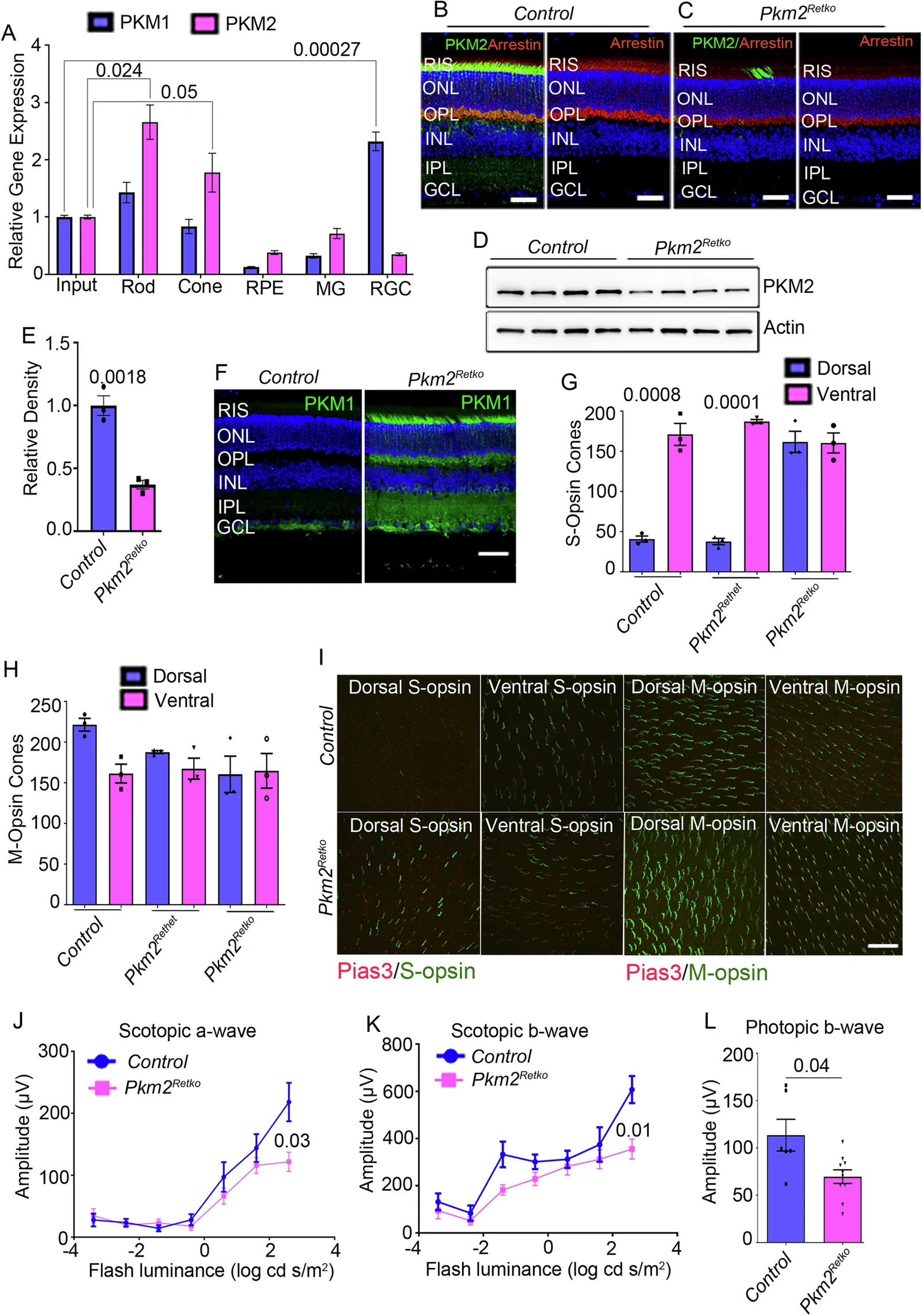
Characterization of *Pkm2*^*Retko*^ mice. TRAP assays were performed using two-month-old mice. TRAP measured PKM1 and PKM2 expression in total retina (input), Rod, Cone, RPE, MG, and RGC by qRT-PCR analysis (**A**). Data are mean ± *SEM* (*n* = *3*). Immunohistochemistry (**B**, **C**) and immunoblot (**D**) were used to assess PKM2 expression in one-month-old *Pkm2*^*Retko*^ mice and littermate controls. Retinal sections were co-stained for PKM2 and arrestin, and panels showing arrestin staining alone are also presented (**B**, **C**). Immunoblotted PKM2 (**D**) was normalized to actin (**E**). Data are mean ± *SEM* (*n* = *4*). PKM1 expression in *Pkm2*^*Retko*^ and littermate controls (**F**). Quantitative analysis of S-opsin positive cones (**G**) and M-opsin positive cones (**H**) in the dorsal and ventral regions of littermate controls, *Pkm2*^*Rethet*^ and *Pkm2*^*Retko*^. Data are mean ± *SEM* (*n* = *3*). Retina flat mounts from littermate control and *Pkm2*^*Retko*^ mice were stained with Pias3, S-opsin, and M-opsin and examined for expression in the dorsal and ventral regions of the retina (**I**). Scotopic a-wave (**J**), scotopic b-wave (**K**), and photopic b-wave (**L**) amplitudes in 3-month-old *Pkm2*^*Retko*^ and littermate controls. Data are mean ± *SEM* (control *n* = *6; Pkm2*^*Retko*^ *n* = *12*).

In contrast, PKM2 shows strong expression in both the outer and inner neuroblastic layers of the retina at P2 (Fig. 2A). This pattern continued through P7 (Fig. 2E). By P7, PKM2 presents in photoreceptors and other regions of the retina (Fig. 2E). Photoreceptor-specific protein, rhodopsin expression starts at P7 (Fig. 2E). At P9, PKM2 expression was primarily observed in photoreceptor inner segments and the outer plexiform layer, with some expression in the inner plexiform and ganglion cell layers (Fig. 2G). This trend continued up to P14 and P17 (Fig. 2K, M). By P21, PKM2 expression is primarily established in the rod inner segments and the outer plexiform layer, with decreased levels in the inner plexiform and ganglion cell layers (Fig. 2O). Overall, our results show that PKM1 expression in the retina begins later (P14) than PKM2 (P2) during postnatal development. To examine the expression of PKM1 and PKM2 in retinal cell types, we isolated mRNA from the entire retina (input), rods, cones, retinal pigment epithelium (RPE), Müller cells, and retinal ganglion cells (RGCs) using Translating Ribosome Affinity Purification (TRAP) followed by qRT-PCR [15]. These results demonstrate that PKM2 is abundantly expressed in rods and cones, compared to RPE, Müller cells, and RGCs. Conversely, PKM1 is predominantly expressed in RGCs (Fig. 3A).

### Retina-specific deletion of PKM2 reveals its role in retinal development

We previously reported studies using rod photoreceptor–specific PKM2 knockout mice generated with rhodopsin-Cre, which is expressed in rods starting around postnatal day 7 [16]. In this study, we deleted PKM2 in the retina (*Pkm2*^*Retko*^) using Cre-recombinase under the control of the Chx10 promoter, a homeobox gene expressed in all retinal progenitor cells [17]. One-month-old *Pkm2*^*Retko*^ mouse retina shows a loss of PKM2 expression in all retinal cells compared to littermate controls (*Pkm2*^*flox/flox*^) (Fig. 3B, C). This was further confirmed with immunoblot analysis (Fig. 3D, E). The residual PKM2 protein expression likely reflects incomplete Chx10-Cre recombination and mosaic deletion in retinal progenitors, resulting in persistence of PKM2 in a subset of retinal neurons. Remaining protein may also derive from non-neuronal retinal cells, such as vascular and immune cells, which are not targeted by Chx10-Cre. Histological examination of the one-month-old retina stained with Hematoxylin and Eosin reveals no significant morphological differences between control, *Pkm2*^*Rethet*^, and *Pkm2*^*Retko*^ mice (Fig. S1). These observations suggest that the loss of PKM2 during retinal development does not significantly impact the overall structure of the retina.

Exons 9 and 10 of the *PKM* gene are equal in length, with exon 9 producing PKM1 and exon 10 producing PKM2 [2]. Exon 9 must be suppressed during splicing to ensure exon 10 is included in the final PKM2 transcript [2]. We previously reported that PKM2 is expressed in photoreceptor cells, whereas PKM1 is exclusively localized to inner retinal layers [18]. In general, PKM2 suppresses PKM1 expression, and loss of PKM2 leads to increased PKM1 expression in photoreceptor cells [18]. Consistent with the splicing data, the loss of PKM2 in the retina led to increased PKM1 expression in rod photoreceptors and enhanced PKM1 staining in the inner plexiform layer (Fig. 3F).

The monomer of PKM1 is both a kinase and a cytosolic thyroid hormone-binding protein [19, 20]. During early mouse development, thyroid hormone (TH) regulates cone spectral identity through its receptor TRβ2 by repressing S-opsin and activating M-opsin expression, thereby establishing the dorsal–ventral patterning of cone opsins. S-opsin–positive cones are more abundant in the ventral part of the retina compared to the dorsal part. Conversely, M-opsin–positive cones are present in both areas, with a slightly higher concentration in the dorsal retina [21]. Increased PKM1 expression in photoreceptor cells lacking PKM2 suggests a possible link to thyroid hormone signaling. Since PKM1 is a thyroid hormone–binding protein that could influence this pathway, we hypothesized that increased PKM1 might modify thyroid hormone signaling and alter the expression of S-opsin and M-opsin gradient in *Pkm2*^*Retko*^ mice. We found the S-opsin gradient (S-opsin positive cones) was increased in the dorsal region in *Pkm2*^*Retko*^ mice, but the M-opsin (M-opsin positive cones) gradient was not altered (Figs. 3G, H, and S2, S3). There was no significant difference in the expression of S-opsin-positive cones in the dorsal region between *Pkm2*^*Rethet*^ and control littermates (Fig. 3G). Transgenic mouse models with mutations in the *TRβ* gene exhibit altered expression of S- and M-opsins [22]. Furthermore, protein inhibitor of activated STAT3 (Pias3) has been shown to function in mouse cone photoreceptors by promoting M-opsin expression and suppressing S-opsin expression, with transcription factors Trβ2 and Rxrγ mediating the preferential expression of Pias3 in M cones [23, 24]. We observed Pias3 expression in the dorsal region of the *Pkm2*^*Retko*^ mouse retina but not in the dorsal region of the control retinas (Figs. 3I and S4). These experiments suggest that either the loss of PKM2 or the increase in PKM1 expression could regulate Pias3 expression and TH signaling. At 3 months of age, *Pkm2*^*Retko*^ mice showed significantly reduced scotopic a-wave and b-wave amplitudes at the highest light intensity compared to control mice, and photopic b-wave amplitudes were also significantly reduced (Fig. 3J–L).

### Biochemical characterization of *Pkm2*^*retko*^ mice

**Fig. 4:**
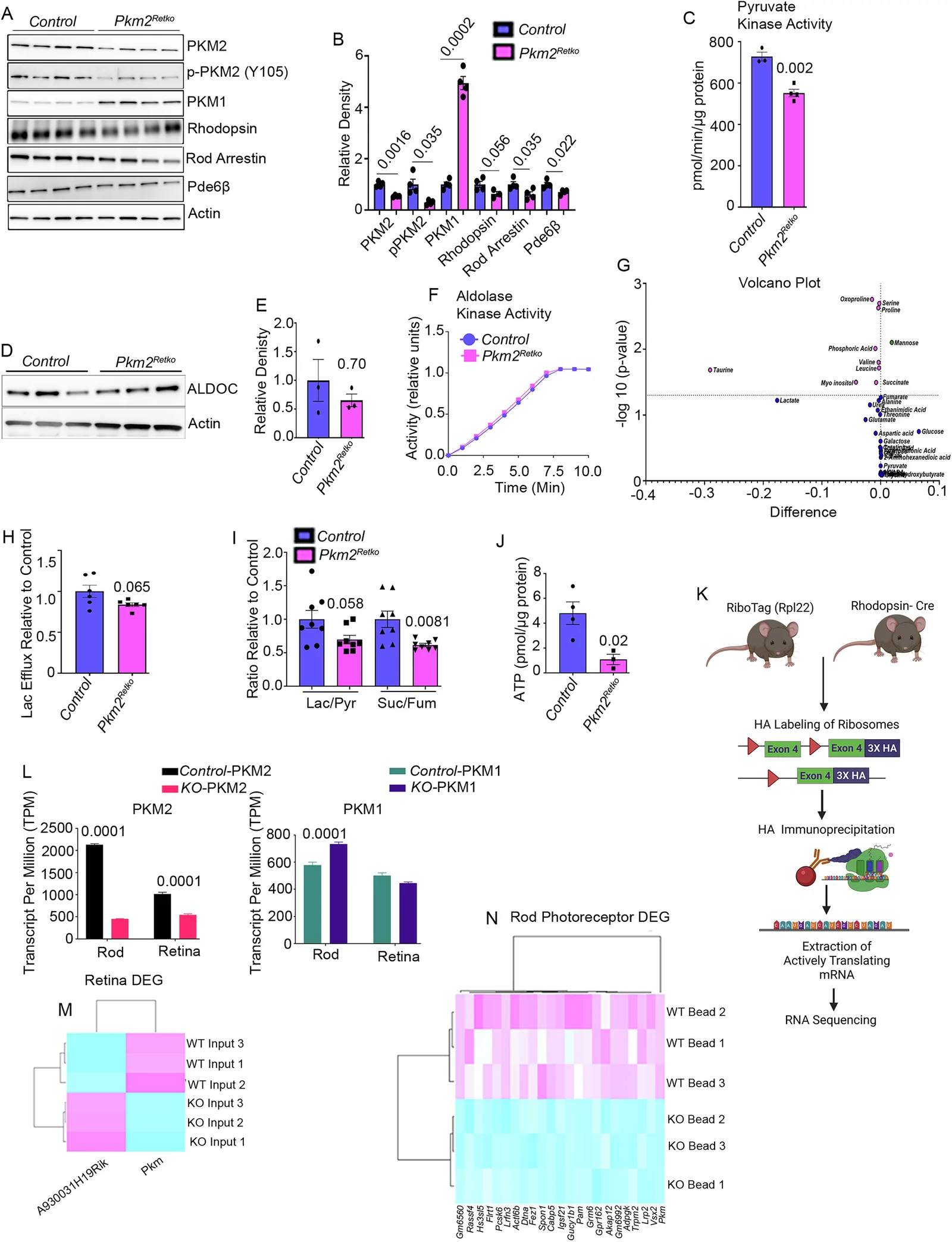
Biochemical and metabolic characterization of *Pkm2*^*Retko*^ mice. Retinal proteins from one-month-old *Pkm2*^*Retko*^ and littermate controls were immunoblotted with PKM2, pPKM2, PKM1, rhodopsin, rod arrestin, pde6β, and actin (**A**), and their levels were normalized to actin (**B**). Data are mean ± *SEM* (*n* = *4*). Pyruvate kinase activity was measured from *Pkm2*^*Retko*^ and littermate controls (**C**). Data are mean ± *SEM* (*n* = *4*). ALDOC levels (**D**), normalized to actin (**E**), and activity (**F**) were measured from *Pkm2*^*Retko*^ and littermate controls. Data are mean ± *SEM* (*n* = *3 for protein; n* = *5 for activity*). Volcano plot of steady-state metabolites measured by GC–MS in *Pkm2*^*Retko*^ and control retinas (**G**). The x-axis shows mean rank difference (*Pkm2*^*Retko*^ vs control), and the y-axis shows –log10 (p-value). Each point represents a metabolite (*n* = *8*). Positive values indicate higher levels in *Pkm2*^*Retko*^, and negative values indicate lower levels. Ex vivo-retinal explants from 2-month-old *Pkm2*^*Retko*^ and littermate controls were incubated in KRB buffer containing 5 mM glucose and measured lactate release after 30 min (**H**). Data are mean ± *SEM* (*n* = *6*). Lactate/Pyruvate and Succinate/Fumarate ratios were plotted from the steady-state metabolite data (**I**). Data are mean ± *SEM* (control *n* = *6; Pkm2*^*Retko*^ *n* = *8*). ATP levels were measured from retinas of *Pkm2*^*Retko*^ and littermate controls (**J**). Data are mean ± *SEM* (*n* = *4*). Schematic representation of TRAP showing the crossing of Rpl22 mice with rod-specific rhodopsin Cre (**K**). Transcript per million mapped reads (TPM) of PKM2 and PKM1 are measured from the rod and retina of 2-month-old wild-type and *Pkm2*^*Rodko*^ mice (**L**). Heatmaps of differentially expressed genes (DEG) in retina (**M**) and rod photoreceptor cells (**N**) in *Pkm2*^*Retko*^ and littermate controls. Data are mean ± *SEM* (*n* = *3*). Note: The PKM2 and actin blots shown in Fig. 3D and 4A are the same, and the remaining panels were generated from samples collected at the same age. Presenting them in Fig. 4A allows direct comparison of the data.

One-month-old *Pkm2*^*Retko*^ mouse retina shows significantly decreased protein levels of PKM2, pPKM2 (Y105), and increased levels of PKM1 compared to the control mouse retina (Fig. 4A, B). There was a significant decrease in rhodopsin, rod arrestin, and Pde6β levels in the *Pkm2*^*Retko*^ retina compared to the control (Fig. 4A, B). Both PKM1 and PKM2 catalyze the conversion of phosphoenolpyruvate to pyruvate; however, PKM2 is allosterically activated by fructose 1,6-bisphosphate (FBP), and its activity is inhibited by phosphorylation of the tyrosine 105 residue in PKM2 [25]. We found that PKM2 activity is significantly decreased in the *Pkm2*^*Retko*^ mouse retina compared to control littermates (Fig. 4C). FBP is cleaved by the enzyme aldolase into two three-carbon sugar phosphates: glyceraldehyde 3-phosphate (G3P) and dihydroxyacetone phosphate (DHAP) [26], thereby regulating PKM2 activity. Our earlier studies showed that the aldolase C (ALDOC) isoform is expressed in both the retina and aged RPE [14]. However, in the present study, we did not find any difference in ALDOC protein levels (Fig. 4D, E) or activity (Fig. 4F) between the *Pkm2*^*Retko*^ and control retinas.

### Metabolic alterations in *Pkm2*^*Retko*^ mouse retina

We measured the steady-state levels of targeted retinal metabolites (Table S1) [27] in 2-month-old *Pkm2*^*Retko*^ mouse retinas using GC-MS. The metabolite profile shows decreased lactate levels in *Pkm2*^*Retko*^, with no change in pyruvate levels (Fig. 4G). Interestingly, the levels of amino acids, such as leucine, proline, oxoproline (OxoPro), serine, valine, and taurine, are significantly decreased in *Pkm2*^*Retko*^ mouse retinas compared with control retinas (Fig. 4G). All of these amino acids can facilitate the generation of pyruvate. The decreased levels of these amino acids could be compensating for the loss of PKM2-generated pyruvate. The GC-MS data also show decreased levels of myo-inositol, the precursor of phosphoinositides, in the *Pkm2*^*Retko*^ mouse retina compared to the control retina (Fig. 4G). We also found significantly increased levels of mannose in the *Pkm2*^*Retko*^ mouse retina compared to the control retina (Fig. 4G).

The lactate efflux assay showed a decrease in lactate release from *Pkm2*^*Retko*^ retinas compared to controls; however, the difference was not statistically significant when retinas were provided with glucose (Fig. 4H). Interestingly, in the retinas of *Pkm2*^*Retko*^ mice, both the lactate/pyruvate and succinate/fumarate ratios were significantly decreased (Fig. 4I), indicating a lower reducing potential in the retina, likely within rod photoreceptors (reflecting a decreased NADH/NAD⁺ ratio). Consistent with this, ATP production was also significantly decreased compared to control retinas (Fig. 4J). However, the levels of pyruvate dehydrogenase (PDH) and its phosphorylation remained unchanged between control and *Pkm2*^*Retko*^ mice (Fig. S5).

Quantitative proteomics was performed using multiplexed selected reaction monitoring (SRM) [28]. Retinal proteins were extracted, digested with trypsin, and analyzed by liquid chromatography–tandem mass spectrometry (LC-MS/MS). Targeted quantitative proteomic analysis was then used to measure antioxidant/stress response proteins, β-oxidation enzymes, glycolytic and gluconeogenic enzymes, Krebs cycle enzymes, and housekeeping proteins in the *Pkm2*^*Retko*^ mouse retina, and the results were compared to those from control retinas. Among the antioxidant/stress proteins, levels of thioredoxin (Txn1) and peroxiredoxin 5 (Prdx5) were significantly decreased in *Pkm2*^*Retko*^ retina compared to *control* (Fig. S6A). In the β-oxidation panel, acetyl-CoA dehydrogenase family member 11 (Acad11) and acetyl-CoA thioesterase (Acot13) levels were significantly decreased in *Pkm2*^*Retko*^ retina (Fig. S6B). The glycolysis and gluconeogenesis panel showed that fructose-1,6-bisphosphatase (Fbp1) levels were significantly increased, while PKM2 levels were significantly decreased in *Pkm2*^*Retko*^ retina compared to controls (Fig. S6C). In the Krebs cycle panel, lactate dehydrogenase 2 (Idh2) and succinate dehydrogenase complex iron-sulfur subunit B (Sdhb) were both significantly decreased in *Pkm2*^*Retko*^ retina (Fig. S6D). Finally, the housekeeping protein panel showed a significant reduction in hemoglobin alpha (HbA) levels in *Pkm2*^*Retko*^ retina compared to *control* (Fig. S6E). These results indicate that loss of PKM2 disrupts multiple metabolic pathways, including antioxidant defense, fatty acid oxidation, glycolysis, and mitochondrial metabolism. Together, these changes suggest a broad metabolic imbalance in the *Pkm2*^*Retko*^ retina.

### Pkm2-regulated gene expression in rod photoreceptor cells

TRAP (Fig. 4K) followed by RNA-seq analysis revealed a significant reduction in PKM2 mRNA levels in rods from *Pkm2*^*Rodko*^ mice (Fig. 4L–N). The baseline expression of *Pkm2* is approximately 2130 transcripts per million (TPM), which decreased by approximately 79% to 450 TPM in *Pkm2*^*Rodko*^ rods. *Pkm1* expression, on the other hand, increased ~27% from 578 TPM to 734 TPM in rods (Fig. 4L). Since PKM2 is mainly expressed in rods, the TPM levels of PKM2 are significantly lower in the retina (Fig. 4L). RNA sequencing yielded reads for ~16,000 genes in our retina samples and 13,000 genes in our rod-enriched samples. GO Analysis (http://geneontology.org/) of the Top 500 enriched genes after Rod-TRAP revealed transcripts involved in processes such as visual perception, photoreceptor function and development, and ciliary assembly. Further validating Rod-TRAP as a suitable method to enrich for Rod-specific transcripts, similar to the data published earlier [29]. A comparison of retinal mRNA samples identified two differentially regulated genes: *A930031H19Rik* and *Pkm* (Fig. 4M). A comparison between rod-enriched mRNA samples of *Pkm2*^*WT*^ and *Pkm2*^*Rodko*^ identified 23 differentially down-regulated genes in *Pkm2*^*Rodko*^ compared to *Pkm2*^*WT*^ rod samples (Fig. 4N). These genes include *Gm6560* (pseudogene of *Pkm*), *Rassf4* (Ras association domain family member 4), *Hs3sl5* (heparan sulfate-glucosamine 3-sulfotransferase 6), *Flrt1* gene (fibronectin leucine rich transmembrane protein 1), *Pcsk6* (proprotein convertase subtilisin/kexin type 6), *Lrfn3* (leucine-rich repeat and fibronectin type III domain containing 3), *Actl6b* gene (actin-like 6B), *Dtna* (dystrobrevin alpha), *Fez1* (fasciculation and elongation protein zeta 1), *Spon1* (spondin 1, also known as F-spondin), *Cabp5* (calcium-binding protein 5), *Igsf21* (immunoglobulin superfamily member 21), *Gucy1b1* (guanylate cyclase 1 soluble subunit beta 1), *Pam* (peptidylglycine alpha-amidating monooxygenase), *Grm6* (glutamate receptor, metabotropic 6), *Gpr162* (rhodopsin-like class A G-protein-coupled receptor 162), *Akap12* (A-kinase anchoring protein 12), *Gm6992* (pseudogene corresponding to the muscle-specific pyruvate kinase), *Adpgk* (ADP-dependent glucokinase), *Trpm2* (transient receptor potential cation channel, subfamily M, member 2), *Lrp2* (Low-Density Lipoprotein Receptor-Related Protein 2), *Vsx2* (Visual System Homeobox 2) and *Pkm* (Pyruvate kinase). We confirmed some of the identified genes by qRT-PCR, further supporting their downregulation in the *Pkm2*^*Rodko*^ mouse retina compared to the *Pkm2*^*WT*^ mouse retina (Fig. S7).

### PKM2 loss in retina and its impact on retinal pigment epithelium (RPE)

**Fig. 5:**
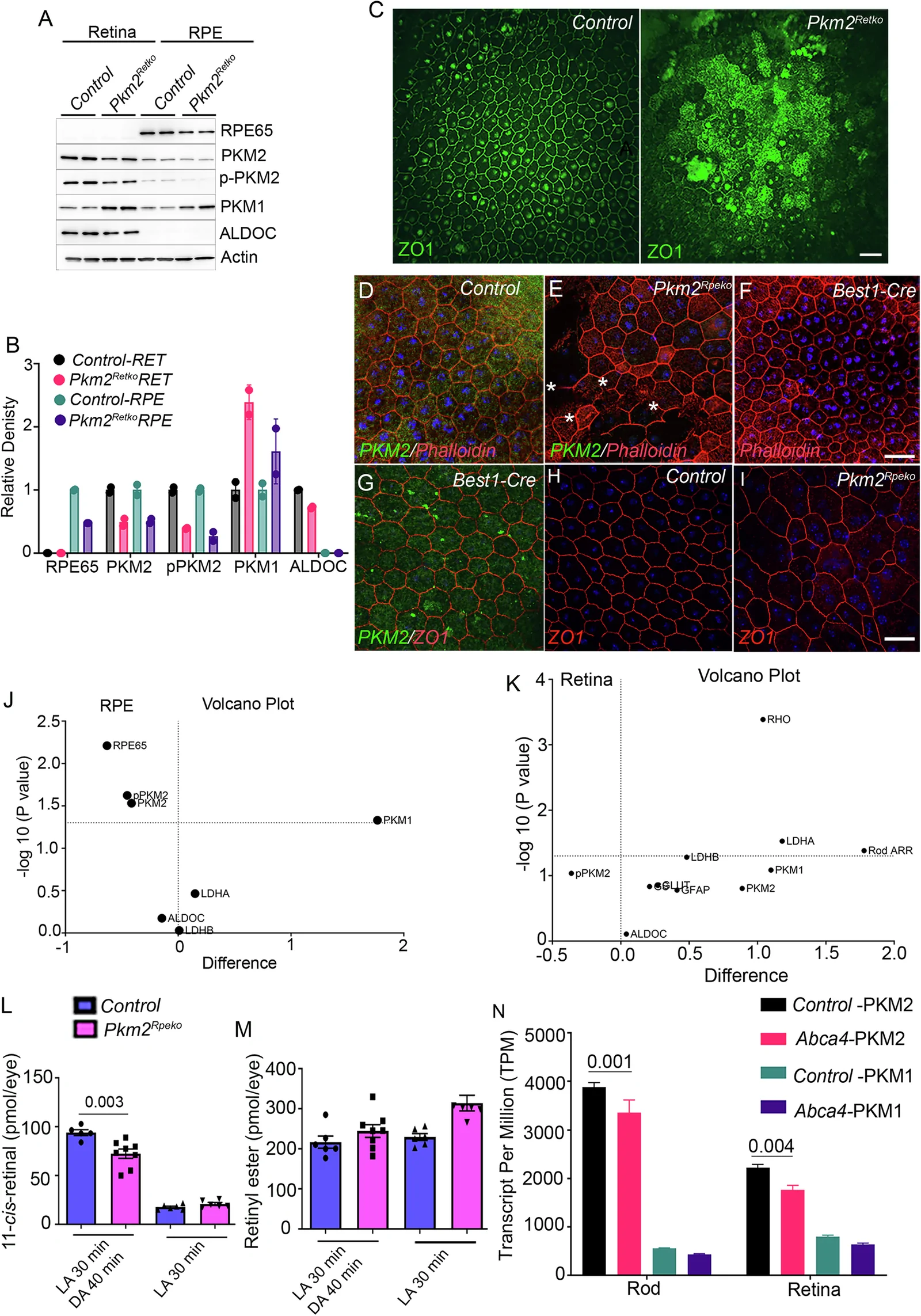
Comparative analysis of protein levels in the retina and RPE cells of *Pkm2*^*Retko*^ mice. Retina and RPE tissues from two-month-old control and *Pkm2*^*Rodko*^ mice were isolated, and proteins were immunoblotted with antibodies against RPE65, PKM2, pPKM2, PKM1, ALDOC, and actin (**A**). Protein levels were quantified and normalized to actin (**B**). Data are mean ± *SEM* (*n* = *2)*. Flat mounts were prepared from five-month-old *Pkm2*^*Retko*^ and control littermates and stained with ZO1 antibody (**C**). Flat mounts from one-month-old control littermates (**D**, **H**), *Pkm2*^*Rpeko*^ (**E**, **I**), and Best1-Cre (**F**, **G**) mice were stained with PKM2 and phalloidin (**D**, **E**), phalloidin alone (**F**), PKM2 and ZO-1 (**G**), or ZO-1 alone (**H**, **I**). *Denotes the loss of the RPE barrier. Volcano plots of protein levels in RPE (**J**) and Retina (**K**) from *Pkm2*^*Rpeko*^ and control littermates. High-performance liquid chromatography (HPLC) was used to analyze retinoid profiles from fully dark-adapted and light-exposed eyes of 5-month-old control and *Pkm2*^*Retko*^ mice. Mice were exposed to bright light (5000 lux for 30 min) and then allowed to dark-adapt for 40 min; control eyes were collected immediately after light exposure in the dark. The levels of 11-*cis*-RAL (**L**) and all-*trans*-REs (**M**) were quantified to assess visual chromophore regeneration. LA, light adaptation; DA, dark adaptation. Data are mean ± *SEM* (control LA, *n* = *6; Pkm2*^*Rpeko*^ LA, *n* = *6*; control DA, *n* = *8*; *Pkm2*^*Rpeko*^, *n* = *6*). Transcript per million mapped reads (TPM) of PKM2 and PKM1 are measured from the rod and retina of two-month-old control and *Abca4*^*Ko*^ mice (**N**). Data are mean ± *SEM* (*n* = *3*).

We prepared RPE and retina from *Pkm2*^*Retko*^ and control mice and examined the levels of RPE65, PKM2, pPKM2, PKM1, and ALDOC. We found that RPE65 levels are decreased in *Pkm2*^*Retko*^ mouse RPE compared with control mouse RPE (Fig. 5A, B). The levels of PKM2 and pPKM2 decreased, while PKM1 levels increased in the RPE, and these results are similar to those observed in the retina of *Pkm2*^*Retko*^ mice compared to control mice (Fig. 5A, B). ALDOC expression was absent in the RPE, but its levels were lower in the retina of *Pkm2*^*Retko*^ mice than in control mice (Fig. 5A, B). Flat mounts prepared from 5-month-old *Pkm2*^*Retko*^ and control mouse RPE, stained with the tight junction protein, Zonula occludens-1 (ZO-1), show uniform ZO-1 staining along the borders of the RPE in control mice. The ZO1 staining was completely disorganized in *the Pkm2*^*Retko*^ mouse RPE (Fig. 5C). These observations suggest that retinal PKM2 may regulate RPE cell integrity.

### Consequences of RPE-specific PKM2 loss on RPE and retinal integrity

We deleted PKM2 in the RPE (*Pkm2*^*Rpeko*^) employing a Bestrophin 1-Cre (Best1-Cre) [30]. RPE flat mounts from one-month-old *Pkm2*^*Rpeko*^ and control mice were stained with PKM2 and phalloidin. The results show that PKM2 expression is decreased in *Pkm2*^*Rpeko*^ RPE compared to control RPE (Fig. 5D, E). Phalloidin staining revealed the loss of RPE cell integrity in *Pkm2*^*Rpeko*^ RPE (Fig. 5E). In contrast, the RPE integrity was well preserved in the control RPE (Fig. 5D). ZO-1 staining of RPE flat mounts showed a regular cobblestone pattern with well-defined cell borders in control mice (Fig. 5H). In *Pkm2*^*Rpeko*^ mice, the RPE monolayer remained intact, but some cells appeared enlarged and showed mild irregularities in cell shape and junctional organization compared with controls (Fig. 5I). To rule out Cre-associated toxicity, Best1-Cre RPE flat mounts were stained with phalloidin (Fig. 5F), PKM2 (Fig. 5G), and the tight junction marker ZO-1 (Fig. 5G), all of which showed intact RPE structure and normal PKM2 expression similar to control (Fig. 5D, H), indicating that the defects observed in *Pkm2*^*Rpeko*^ RPE result from PKM2 deletion rather than Cre expression.

To validate these observations at the protein level, we performed immunoblot analysis of RPE lysates. We found significantly decreased levels of PKM2 and pPKM2, and increased PKM1 levels in *Pkm2*^*Rpeko*^ mouse RPE compared with control RPE (Figs. 5J and S8A, B). Interestingly, the loss of PKM2 in RPE resulted in a significantly decreased level of RPE65 protein in *Pkm2*^*Rpeko*^ compared to control RPE (Figs. 5J and S8A, B). Retinal proteins from *Pkm2*^*Rpeko*^ and control mice were examined for levels of PKM2, pPKM2, PKM1, LDHA, LDHB, ALDOC, Glut1, GFAP, GS, Rhodopsin, and rod arrestin. We found increased levels of rhodopsin, LDHA, and rod arrestin in *Pkm2*^*Rpeko*^ mouse retinas compared to control mouse retinas (Figs. 5K and S8C, D).

RPE65 is an isomerohydrolase in the retinoid visual cycle that converts all-trans-retinyl esters to 11-*cis*-retinol, a key step in regenerating 11-*cis*-retinal (11-*cis*-RAL) required for phototransduction in photoreceptor cells [31]. Retinyl esters, formed by esterifying all-*trans*-retinol in the RPE, serve as substrates for RPE65. To determine whether loss of PKM2 in the RPE affects 11-*cis*-RAL regeneration, control and *Pkm2*^*Rpeko*^ mice were exposed to bright light (5000 lux for 30 min) and then dark-adapted for 40 min. Control mice were exposed to the same light conditions, but their eyes were collected immediately in the dark. Retinoid profiles from whole eyes were analyzed by HPLC to measure the levels of 11-*cis*-RAL and retinyl esters. After 40 min of dark adaptation, 11-cis-RAL levels were significantly lower in *Pkm2*^*Rpeko*^ mice than in the controls (Fig. 5L). In the control (no dark adaptation) group, both genotypes exhibited decreased 11-*cis*-RAL levels (~17% of baseline) (Fig. 5L). Retinal ester (All-trans-RE) levels were slightly higher in *Pkm2*^*Rpeko*^ mice than in control mice; however, the difference was not statistically significant (Fig. 5M). These results suggest that PKM2 modulates retinoid metabolism in the RPE, possibly by regulating RPE65 expression in vivo. Furthermore, ERG recordings showed a trend toward reduced rod function in 2.5-month-old *Pkm2*^*Rpeko*^ mice compared with control mice, while cone function remained unchanged (Fig. S9A–C).

### Rod-specific PKM2 deletion promotes early retinal degenerative changes in Abca4 knockout mice

ABCA4 encodes an ATP-binding cassette transporter localized in photoreceptor outer segments, where it plays a key role in clearing all-trans-retinal derivatives from photoreceptor disc membranes [32]. Abca4 knockout (*Abca4*^*Ko*^) mice are a widely used model for studying Stargardt disease type 1 (STGD1) [32]. In *Abca4*^*Ko*^ mice, the RPE and photoreceptor cells exhibit several hallmark signs of degeneration. In the RPE of *Abca4*^*Ko*^ mice, there is a progressive accumulation of lipofuscin and A2E, derived from bisretinoids, due to impaired clearance of all-trans-retinal. This leads to oxidative stress and RPE dysfunction, which contributes to cell death over time. In photoreceptor cells, especially rods, there is a slow but steady loss of outer segment structure and retinal thinning, which becomes more apparent with age [33]. Although rod photoreceptors are primarily affected, cone degeneration can follow as a secondary consequence. The combination of RPE dysfunction and photoreceptor loss leads to a decline in visual function, mirroring the pathology seen in human diseases such as Stargardt macular dystrophy and aspects of dry AMD [33].

Comparison of C57Bl6 (wild-type) mouse retina with *Abca4*^*Ko*^ retina shows the absence of ABCA4 expression in *Abca4*^*Ko*^ mice compared to control mice (Fig. S10). Rod-TRAP analysis reveals that PKM2 expression decreases in rods with ABCA4 loss, whereas PKM1 expression does not decrease, suggesting a role for PKM2 in AMD (Fig. 5N). The baseline expression of *Pkm2* is approximately 3887 TPM and decreased by approximately 13.5% to 3359 TPM in *Abca4*^*Ko*^ rods. *Pkm1* expression, on the other hand, decreased ~22% from 555 TPM to 436 TPM in rods (Fig. 5N). Since PKM2 is mainly expressed in rods, the TPM levels of PKM2 in control and ABCA4 are significantly lower in the retina compared to rods (Fig. 5N).

In five-month-old *Abca4*^*Ko*^ mice, levels of cone arrestin and PKM1 are significantly lower than in wild-type mice (Fig. S10). No significant differences were observed in the levels of rhodopsin, Pde6β, LDHA, LDHB, rod arrestin, and GFAP between wild-type and *Abca4*^*Ko*^ mouse retina (Fig. S10). RNA-seq analysis revealed 96 differentially expressed genes in the retina and 348 in rods, primarily associated with chemokine signaling (Table S2).

**Fig. 6:**
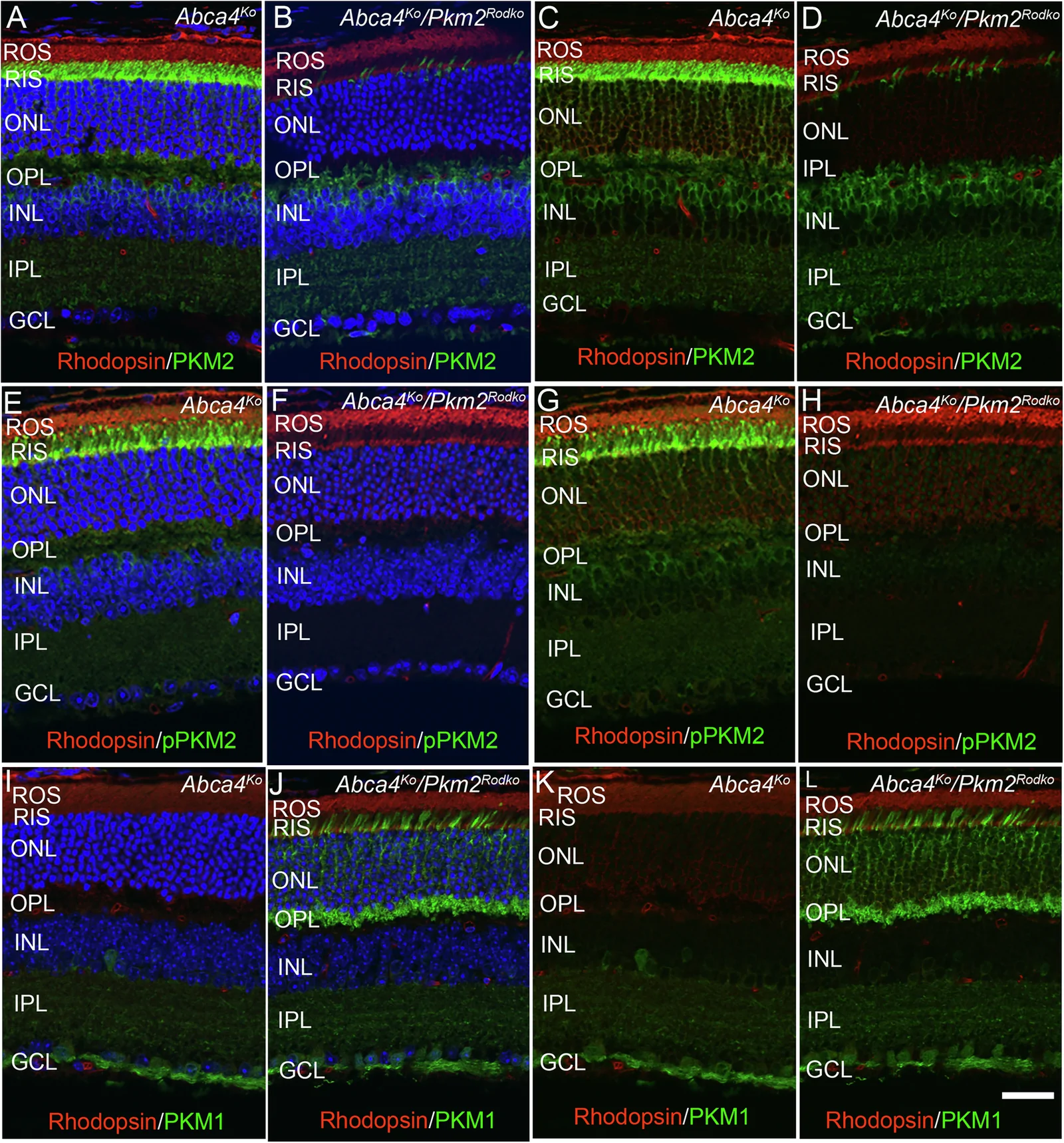
Expression of PKM2, pPKM2, PKM1, and Rhodopsin in *Abca4*^*Ko*^ and *Abca4/Pkm2*^*Rodko*^ mice. Retinal sections from 4-month-old *Abca4*^*Ko*^ (**A**, **C**, **E**, **G**, **I**, **K**) and *Abca4/Pkm2*^*Rodko*^ (**B**, **D**, **F**, **H**, **J**, **L**) mice were stained for rhodopsin and PKM2 (**A**–**D**), rhodopsin and pPKM2 (**E**–**H**), and rhodopsin and PKM1 (**I**–**L**). **C**, **D**, **G**, **H**, **K**, **L** show images without DAPI. Scale bar = 50 µm.

We bred rod-specific PKM2 knockout mice (*Pkm2*^*Rodko*^) with Abca4 knockout (*Abca4*^*Ko*^) mice to generate mice with rod-specific deletion of PKM2 in the *Abca4*^*Ko*^ background (*Abca4*^*Ko*^*/Pkm2*^*Rodko*^) and control mice (*Abca4*^*Ko*^). Immunohistochemistry confirmed the loss of PKM2 (Fig. 6A–D) and its phosphorylation (Fig. 6E–H) in 4-month-old *Abca4*^*Ko*^*/Pkm2*^*Rodko*^ mice compared with *Abca4*^*ko*^ mice. The residual PKM2 staining observed in the rod inner segment region of *Abca4*^*Ko*^*/Pkm2*^*Rodko*^ retinas likely originates from cone photoreceptors (Fig. 6B, D). Previous studies have shown that PKM1 is expressed in the inner plexiform and ganglion cell layers, while PKM2 is predominantly expressed in photoreceptor cells [18]. Loss of PKM2 in rod photoreceptors leads to compensatory upregulation of PKM1. In the *Abca4*^*Ko*^*/Pkm2*^*Rodko*^ retina, PKM1 expression is strongly increased in the rod inner segments (RIS), outer nuclear layer (ONL), and outer plexiform layer (OPL) (Fig. 6I–L). This pattern differs from that observed in rod-specific PKM2 knockout mice, where PKM1 expression is mainly detected in the RIS and OPL, with only limited expression in the ONL [16]. The PKM1 staining pattern in *Abca4*^*Ko*^*/Pkm2*^*Rodko*^ retina suggests altered PKM1 localization in these mice.

**Fig. 7:**
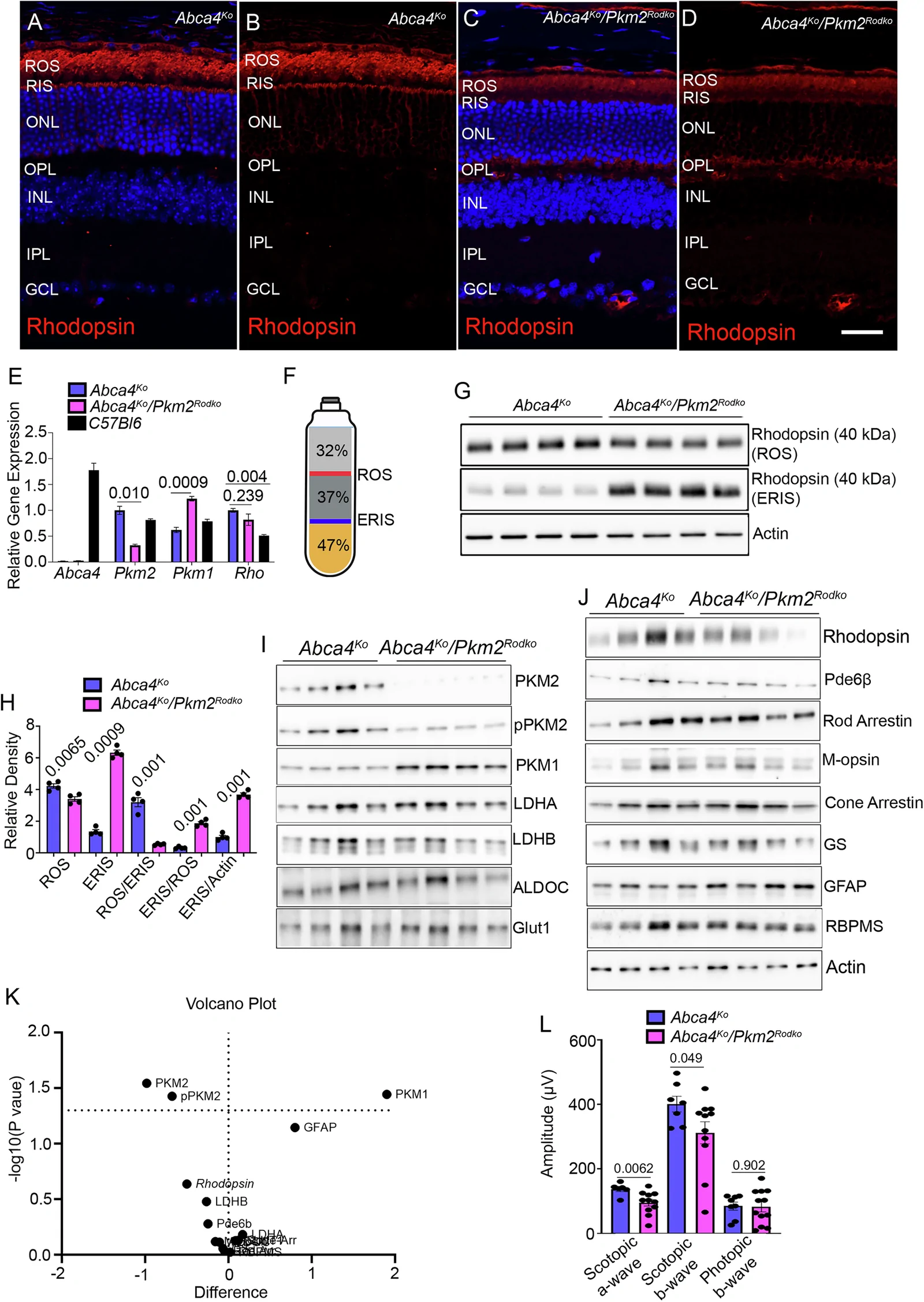
Rhodopsin expression, localization, gene expression, and functional characterization in *Abca4*^*Ko*^ and *Abca4/Pkm2*^*Rodko*^ mice. Retinal sections from 4-month-old *Abca4*^*Ko*^ (**A**, **B**) and *Abca4/Pkm2*^*Rodko*^ (**C**, **D**) mice were stained for rhodopsin and counterstained with DAPI. **B**, **D** show rhodopsin staining alone. Scale bar = 50 µm. Equal amounts of retinal mRNA from three independent 3-month-old C57Bl6, *Abca4*^*Ko*^ and *Abca4/Pkm2*^*Rodko*^ mice were used for qRT-PCR to assess the expression of *Abca4*, *Pkm2, Pkm1, Rho*, and the results were normalized to actin levels (**E**). Rod outer segments (ROS) and rod inner segment–enriched fractions (ERIS) were prepared from 2-month-old *Abca4*^*Ko*^ and *Abca4/Pkm2*^*Rodko*^ mice using discontinuous sucrose gradient centrifugation (**F**). Isolated ROS and ERIS fractions from four independent preparations were analyzed by immunoblotting for rhodopsin and actin (**G**). Quantitative analysis is shown as ROS, ERIS, ROS/ERIS, ERIS/ROS, and ERIS normalized to actin (**H**). Data are presented as mean ± *SEM (n* = *4)*. Retinal proteins from *Abca4*^*Ko*^ and *Abca4*^*Ko*^*/Pkm2*^*Retko*^ mice were immunoblotted with PKM2, pPKM2, PKM1, LDHA, LDHB, ALDOC, Glut1 (**I**), rhodopsin, pde6β, rod arrestin, M-opsin, cone-arrestin, GS, GFAP, RBPMS, and actin (**J**). Volcano plots of protein levels in the retina of *Abca4*^*Ko*^ and *Abca4*^*Ko*^*/Pkm2*^*Retko*^ mice (**K**). Each point represents an individual protein quantified from four biological replicates per group (mean ± *SEM*). Scotopic a-wave, scotopic b-wave, and photopic b-wave analyses were performed on 3-month-old *Abca4*^*Ko*^ and *Abca4*^*Ko*^*/Pkm2*^*Retko*^ (**L**). Data are mean ± *SEM (n* = *11)*.

In addition, rhodopsin levels in the outer segments appeared reduced in 4-month-old A*bca4*^*Ko*^*/Pkm2*^*Rodko*^ retinas compared with *Abca4*^*ko*^ mice (Fig. 7A–D). In *Abca4*^*ko*^ mice, rhodopsin was properly localized to the outer segments (Fig. 7A, B), whereas in A*bca4*^*Ko*^*/Pkm2*^*Rodko*^ retinas, rhodopsin immunoreactivity was detected in the outer segments, inner segments, and prominently in the outer plexiform layer (OPL) (Fig. 7C, D). These observations suggest that rhodopsin is mislocalized in the A*bca4*^*Ko*^*/Pkm2*^*Rodko*^ retina.

PKM2 can function as a transcriptional co-activator [9]. To determine whether loss of PKM2 in the *Abca4*^*ko*^ retina affects rhodopsin gene expression, we measured mRNA levels of *Abca4, Pkm2, Pkm1*, and *Rhodopsin* in *Abca4*^*Ko*^*/Pkm2*^*Rodko*^, *Abca4*^*ko*^, and C57Bl6 mouse retinas. Consistent with the immunohistochemistry results, PKM2 mRNA was significantly reduced, and PKM1 expression was increased in 3-month-old *Abca4*^*Ko*^*/Pkm2*^*Rodko*^ retinas compared with *Abca4*^*ko*^ retinas (Fig. 7E). *Abca4* expression was absent in both *Abca4*^*ko*^ and *Abca4*^*Ko*^*/Pkm2*^*Rodko*^ retinas relative to C57BL6 controls (Fig. 7E). However, rhodopsin mRNA levels were similar between the two genotypes (Fig. 7E). In contrast, rhodopsin expression was elevated in both *Abca4*^*ko*^ and *Abca4*^*Ko*^*/Pkm2*^*Rodko*^ retinas compared with C57BL/6 control retinas (Fig. 7E). These findings suggest that Abca4 deficiency alters photoreceptor gene expression. We therefore suspected that the reduced rhodopsin levels observed in *Abca4*^*Ko*^*/Pkm2*^*Rodko*^ retinas may result from protein degradation or mislocalization rather than decreased transcription.

To evaluate rhodopsin mislocalization, rod outer segments (ROS) and enriched rod inner segments (ERIS) were isolated from retinas of 2-month-old *Abca4*^*ko*^ and *Abca4*^*Ko*^*/Pkm2*^*Rodko*^ mice using discontinuous sucrose gradient centrifugation (Fig. 7F). This approach yields highly purified ROS fractions and ERIS fractions that contain rod inner segments together with other retinal cellular components. The purity of these fractions has been previously validated using markers specific for RPE, outer segments, and inner segments [34]. Immunoblot analysis revealed a reduction of rhodopsin in the ROS fraction and a corresponding increase in the ERIS fraction from *Abca4*^*Ko*^*/Pkm2*^*Rodko*^ mice compared with *Abca4*^*ko*^ mice (Fig. 7G, H). When rhodopsin levels in the ERIS fraction were normalized to ROS and actin, a significant increase was observed in ERIS from *Abca4*^*Ko*^*/Pkm2*^*Rodko*^ retinas relative to *Abca4*^*ko*^ retinas (Fig. 7G, H), consistent with mislocalization of rhodopsin to the inner segments, indicating that PKM2 is essential for proper rhodopsin targeting to outer segments in *Abca4*^*ko*^ mice.

Consistent with the immunohistochemistry results, the levels of PKM2 and phosphorylated PKM2 were significantly lower in *Abca4*^*Ko*^*/Pkm2*^*Rodko*^ mice compared with *Abca4*^*Ko*^ mouse retinas, whereas PKM1 levels were significantly higher in *Abca4*^*Ko*^*/Pkm2*^*Rodko*^ mice (Fig. 7I–K). However, no significant differences were observed in the levels of LDHA, LDHB, ALDOC, Glut1, PDE6β, rod arrestin, M-opsin, cone arrestin, GS, or GFAP between the two groups (Fig. 7I–K). The rhodopsin levels in the retina appeared lower in *Abca4*^*Ko*^*/Pkm2*^*Rodko*^ mice, but this difference was not statistically significant compared with *Abca4*^*Ko*^ retinas. Although total rhodopsin levels were comparable between the two genotypes, fractionation of rod outer segments (ROS) and enriched rod inner segments (ERIS) showed mislocalization of opsin to the inner segment in *Abca4*^*Ko*^*/Pkm2*^*Rodko*^ mice. At saturated light intensity, scotopic a- and b-wave amplitudes were significantly reduced in 3-month-old *Abca4*^*Ko*^*/Pkm2*^*Rodko*^ mice compared with *Abca4*^*Ko*^ (Fig. 7L), whereas no differences were observed at lower light intensities (Fig. S9D, E). Photopic b-wave amplitudes were not significantly different between the two groups (Fig. 7L).

**Fig. 8:**
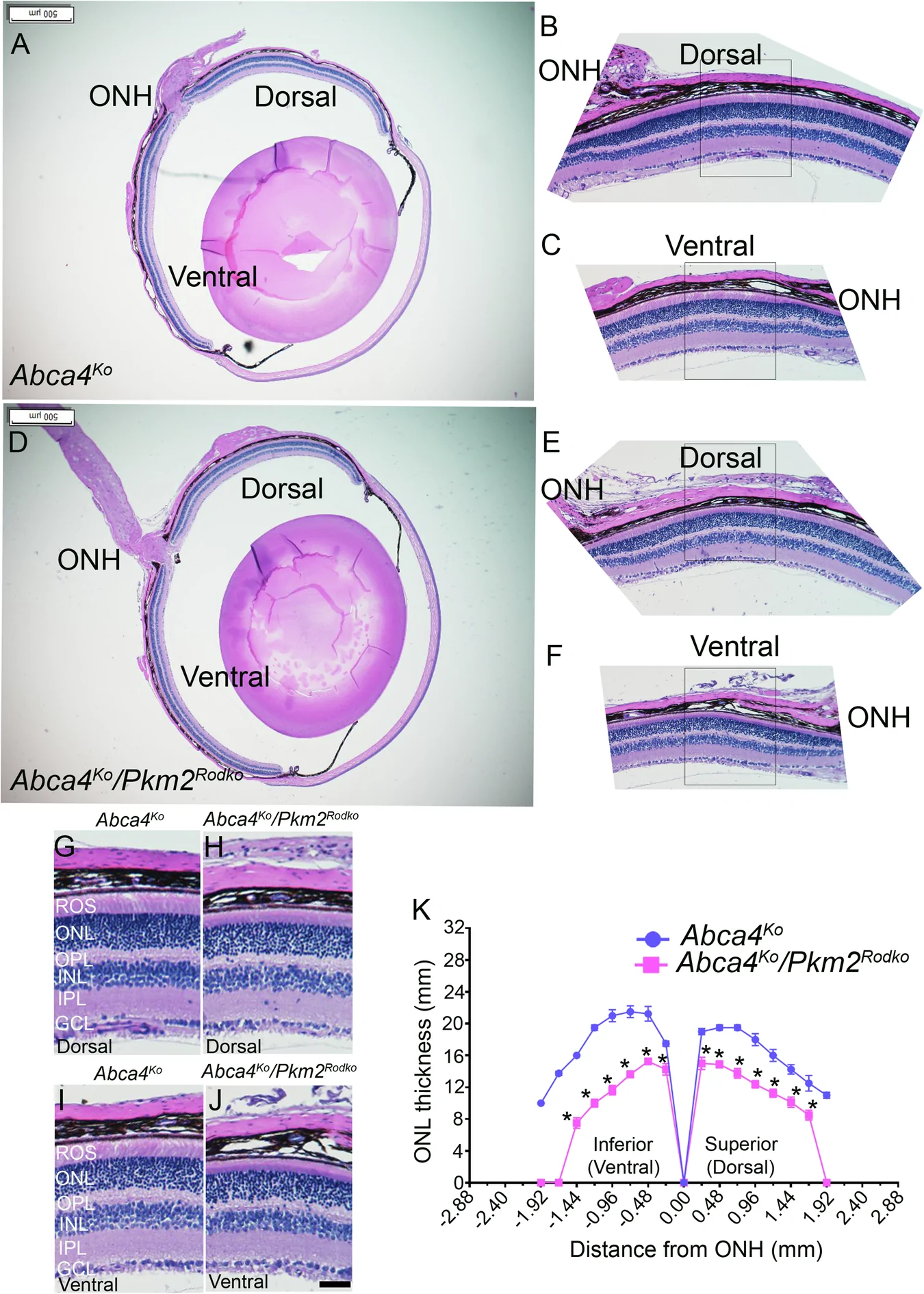
Structural analysis of retinas from *Abca4*^*Ko*^*/Pkm2*^*Rodko*^ mice. Representative whole-eye H&E-stained sections from 4-month-old *Abca4*^*Ko*^ and *Abca4*^*Ko*^*/Pkm2*^*Rodko*^ mice showing overall retinal structure. Dorsal and ventral retinal regions and the optic nerve head (ONH) are indicated (**A**, **D**). Higher magnification images of dorsal and ventral retinal regions from *Abca4*^*Ko*^ and *Abca4*^*Ko*^*/Pkm2*^*Rodko*^ mice (**B**, **C**, **E**, **F**). Boxed regions (**B**, **C**, **E**, **F**) indicate the areas shown at higher magnification in (**G**–**J**), which present representative H&E-stained retinal sections from the dorsal and ventral regions of *Abca4*^*Ko*^ and *Abca4*^*Ko*^*/Pkm2*^*Rodko*^ retina. Quantification of ONL thickness presented as a spider graph based on measurements taken at 0.24 mm intervals along the vertical meridian passing through the optic nerve head (ONH) in dorsal and ventral retinal regions of *Abca4*^*Ko*^ and *Abca4*^*Ko*^*/Pkm2*^*Rodko*^ mice (**K**). Data are mean ± SEM (*Abca4*^*Ko*^, *n* = *8*; *Abca4*^*Ko*^*/Pkm2*^*Rodko*^, *n* = *16*). *p < 0.0001. Scale bar = 50 µm.

Histological analysis of retinal sections from 4-month-old *Abca4*^*Ko*^ and *Abca4*^*Ko*^*/Pkm2*^*Rodko*^ mice demonstrated accelerated retinal degeneration in the double knockout animals (Fig. 8A–J). H&E-stained whole-eye sections showed marked retinal thinning in *Abca4*^*Ko*^*/Pkm2*^*Rodko*^ mice compared with *Abca4*^*Ko*^ controls, particularly in the ventral retina (Fig. 8A–F). Boxed regions in the low-magnification images (Fig. 8B, C, E, F) indicate the corresponding dorsal and ventral retinal areas selected for higher magnification comparison between genotypes (Fig. 8G–J). Higher magnification images showed a substantial reduction in outer nuclear layer (ONL) thickness in *Abca4*^*Ko*^*/Pkm2*^*Rodko*^ mice, indicating enhanced photoreceptor loss following rod-specific deletion of PKM2 in the Abca4-deficient background. Quantitative analysis of ONL thickness at 0.24 mm intervals from the optic nerve head (ONH) along the vertical meridian confirmed significant thinning in both the superior (dorsal) and inferior (ventral) retina of *Abca4*^*Ko*^*/Pkm2*^*Rodko*^ mice, with degeneration being more pronounced in the ventral retina (Fig. 8K).

To determine whether the loss of PKM2 in the RPE of *Abca4*^*Ko*^ mice affects the RPE and retina, we bred RPE-specific PKM2 knockout mice (*Pkm2*^*Rpeko*^) with *Abca4* knockout (*Abca4*^*Ko*^) mice to generate mice with RPE-specific deletion of PKM2 on the *Abca4*^*Ko*^ background (*Abca4*^*Ko*^*/Pkm2*^*Rpeko*^) and control mice (*Abca4*^*ko*^). Interestingly, at two months of age, we found that PKM2 was absent and PKM1 levels were increased in the *Abca4*^*Ko*^*/Pkm2*^*Rpeko*^ mouse RPE compared with the *Abca4*^*Ko*^ mouse RPE (Fig. S11). Similar findings were observed in the retina of *Abca4*^*Ko*^*/Pkm2*^*Rpeko*^ mice (Fig. S11). The pPKM2 levels further confirm the absence of PKM2 and its decreased levels in the retina and RPE (Fig. S11). However, there was no difference in the levels of ALDOC, LDHA, LDHB, rhodopsin, Pde6β, rod arrestin, M-opsin, cone arrestin, and GS in the retina between *Abca4*^*Ko*^ *and Abca4*^*Ko*^*/Pkm2*^*Rpeko*^ mice (Fig. S11). In the RPE, there was no difference in LDHB or occludin levels between *Abca4*^*Ko*^ and *Abca4*^*Ko*^*/Pkm2*^*Rpeko*^ mice; however, in one sample, RPE65 was undetectable in *Abca4*^*Ko*^*/Pkm2*^*Rpeko*^ mice (Fig. S11). Functional assessment revealed a significant reduction in cone function, but not rod function, in 5-month-old *Abca4*^*Ko*^*/Pkm2*^*Rpeko*^ mice compared to *Abca4*^*Ko*^ mice (Fig. S9G–I). At three months of age, we examined additional mice and found that the *Abca4*^*Ko*^*/Pkm2*^*RpeKo*^ RPE showed significantly decreased levels of PKM2, pPKM2, RPE65, and LDHB, and increased levels of PKM1, ALDOC, GFAP, and phosphorylated Akt (Thr308) compared to *Abca4*^*Ko*^ RPE (Fig. S12). Since the RPE functions as the phagocytic center for photoreceptors, the *Abca4*^*Ko*^*/Pkm2*^*RpeKo*^ RPE also exhibited decreased levels of lysosomal markers LAMP1 and LAMP2 and elevated levels of the autophagy marker LC3A/B (Fig. S12), suggesting impairment of the autophagy–endolysosomal pathway.

## Discussion

Photoreceptors are postmitotic neurons that primarily express PKM2, while PKM1 is present at very low levels [18]. During postnatal retinal development, PKM2 expression begins early and increases as photoreceptors differentiate and the retina matures, while PKM1 expression appears later and becomes more prominent in other retinal neurons. This temporal and spatial difference suggests a distinct metabolic role for PKM2 in photoreceptors. Consistent with this, single-cell TRAP data indicate that PKM2 is highly enriched in rods and cones relative to Müller glia and RPE cells, whereas PKM1 is highly enriched in retinal ganglion cells. In rod cells, PKM2 is expressed at about 2130 transcripts per million (TPM), but this level drops by roughly 79% to 450 TPM in *Pkm2*^*Rodko*^ rods. In contrast, PKM1 expression increases by approximately 27%, rising from 578 TPM to 734 TPM. In addition to its role as a metabolic enzyme, PKM2 also functions as a transcriptional co-activator. Consistent with this role, several rod-enriched genes were downregulated in the absence of PKM2, indicating that PKM2 loss may influence gene expression programs associated with photoreceptor function and cellular homeostasis.

Interestingly, deletion of PKM2 in retinal progenitor cells did not affect retinal development; however, the dorsal–ventral patterning of the cone opsin gradient was altered. Whether this alteration is mediated by PKM2 loss or by compensatory upregulation of PKM1 remains unclear. Thyroid hormone (TH) signaling is known to regulate the dorsal-ventral pattern of the cone opsin gradient [22, 35, 36]. The monomer of PKM1 is a TH-binding protein that may interfere with the normal functions of TH. Further studies are needed to investigate the roles of the PKM1/PKM2 axis in TH signaling and their regulation of the cone opsin gradient.

Metabolomic and proteomic analyses of *Pkm2*^*Retko*^ mice revealed substantial metabolic reprogramming and mitochondrial dysfunction. Lactate levels decreased, while pyruvate remained unchanged, resulting in lower lactate/pyruvate and succinate/fumarate ratios, which are indicative of impaired glycolytic flux and mitochondrial redox imbalance. Depletion of amino acids such as leucine, serine, valine, and taurine suggests enhanced catabolism to sustain the TCA cycle. Decreased *myo*-inositol levels indicate impaired phosphoinositide synthesis [37], which may impact membrane signaling and photoreceptor integrity. Mannose is a critical substrate for N-linked glycosylation of proteins that occurs in the endoplasmic reticulum [38]. The observed increase in mannose suggests a potential shift in protein glycosylation pathways, which could impact retinal function and photoreceptor health. Further studies are needed to clarify how elevated mannose levels influence glycosylation and downstream cellular processes in the retina. Collectively, these data suggest that the loss of PKM2 disrupts glycolysis, mitochondrial energy production, redox balance, and biosynthetic pathways critical to retinal function, creating a metabolic environment that may compromise photoreceptor survival.

Although PKM2 expression in the RPE is lower than in photoreceptors, its deletion in the RPE disrupted cellular integrity, decreased RPE65 protein levels, and impaired the regeneration of the visual chromophore 11-*cis-*RAL. PKM2 expression in the RPE increases with age and during NaIO₃-induced degeneration, a model of dry AMD, suggesting an adaptive glycolytic shift in response to stress [14]. Deletion of PKM2 in the RPE resulted in elevated levels of PKM1, rhodopsin, and rod arrestin in the retina, possibly due to impaired RPE phagocytosis. The increase in rhodopsin and arrestin may also result from decreased protein mobilization due to reduced chromophore availability. Our findings also suggest that RPE dysfunction may secondarily suppress photoreceptor aerobic glycolysis and reduce PKM2 expression, as photoreceptors adjust their metabolic activity in response to altered nutrient and retinoid handling and increased oxidative stress associated with impaired RPE function. These findings highlight the role of PKM2 in maintaining RPE homeostasis and metabolic balance in normal physiology.

In disease contexts, PKM2 has divergent roles. Replacement of PKM2 with PKM1 reverses tumorigenic phenotypes [39], while in retinal models, PKM2 ablation in retinitis pigmentosa partially rescues degeneration [40]. Small-molecule activators of PKM2 also show neuroprotective effects under outer retinal stress [41]. In *Abca4*^*Ko*^ mice, PKM1 protein levels decreased, whereas PKM2 protein levels remained unchanged. In contrast, TRAP analysis showed reduced ribosome-associated PKM2 mRNA levels in *Abca4*^*K*o^ rods, indicating reduced translation, whereas PKM1 translation remained unchanged. This difference may be due to differences in protein stability or turnover between the isoforms, allowing PKM2 protein levels to be maintained despite reduced translation. While *Abca4*^*Ko*^ mice maintained normal rhodopsin localization, *Abca4*^*Ko*^/*Pkm2*^*Rodko*^ mice displayed rhodopsin mislocalization to inner segments, retinal thinning, and photoreceptor loss. Mislocalized opsin has been reported to induce endoplasmic reticulum stress in P23H rhodopsin mutant mice [42] and contribute to rod photoreceptor cell death in retinal diseases [43]. Generally, mislocalized opsin is retained in the ER of the photoreceptor inner segment, where it can induce ER stress, activate the unfolded protein response (UPR), and promote ER-associated protein degradation pathways [42, 44]. An alternative possibility is that rhodopsin may be retained in the inner segments without ER entrapment, suggesting that PKM2 could contribute to rhodopsin trafficking to the outer segments. Previous studies have shown that PKM2 and glycolytic metabolism can influence vesicle dynamics and protein trafficking [45]. However, additional studies are needed to directly test this possibility.

Despite unchanged rhodopsin mRNA levels in *Abca4Ko/Pkm2*^*Rodko*^ mice, rhodopsin expression was reduced, suggesting that PKM2 supports both rhodopsin expression and proper trafficking. A recent study reported that key aerobic glycolysis genes, including *Pkm1* and *Pkm2*, are downregulated in mouse models of retinitis pigmentosa during the early stages of the disease [46], supporting the idea that metabolic reprogramming occurs in diseased photoreceptors.

Loss of PKM2 in the *Abca4*^*Ko*^ RPE disrupts cellular metabolism and autophagy. Decreased PKM2 and LDHB, together with increased PKM1, ALDOC, and phosphorylated Akt (Thr308), indicate altered glycolytic regulation and metabolic stress. Decreased RPE65 levels suggest impaired visual cycle activity. In addition, decreased LAMP1 and LAMP2 with elevated LC3A/B reflect defective autophagy–endolysosomal function, which may compromise photoreceptor support and contribute to retinal degeneration.

PKM2 acts not only as a metabolic enzyme but also as a transcriptional co-activator [47]. Its loss can therefore affect both metabolism and gene expression, leading to photoreceptor degeneration. Our previous work demonstrated that deletion of PKM2 in rods decreased PDE6β expression [16], whereas the current study shows that loss of PKM2 in the RPE reduces RPE65 protein levels. These effects may reflect PKM2-dependent regulation of gene expression or increased protein degradation in the absence of PKM2, rather than being solely due to metabolic changes. Further studies are needed to identify PKM2-regulated gene networks in the RPE and to determine how these pathways contribute to retinal homeostasis and degeneration.

**Fig. 9:**
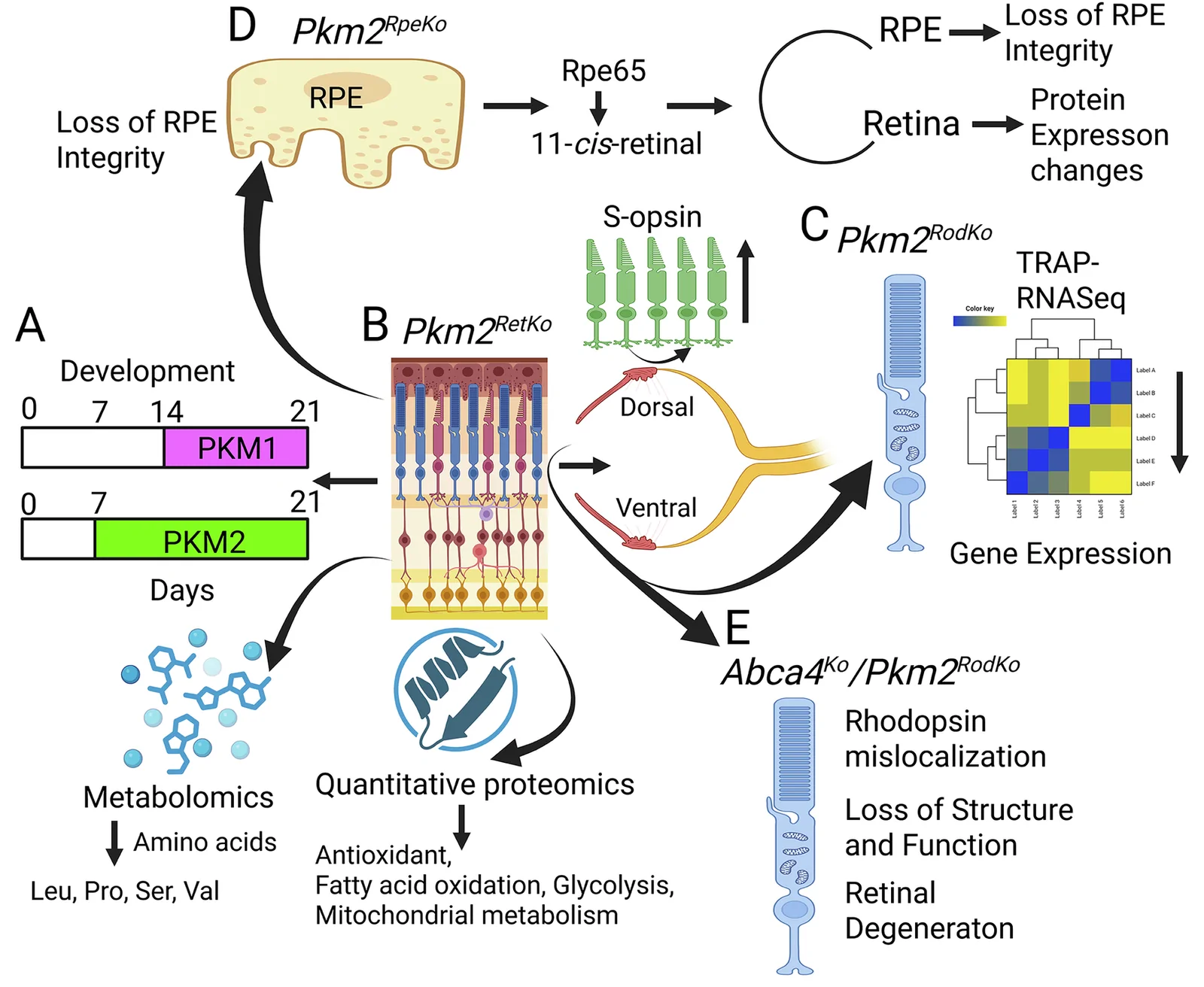
PKM2 regulates retinal metabolism and maintains RPE and photoreceptor integrity. This study examines the role of PKM isoforms in retinal development, metabolism, and photoreceptor function. During retinal development, PKM2 is expressed early, whereas PKM1 expression appears later and is present in different retinal cell types (**A**). Pan-retinal deletion of PKM2 alters the cone opsin gradient, retinal metabolism, including amino acid levels, antioxidant pathways, fatty acid oxidation, glycolysis, and mitochondrial metabolism, and also affects RPE cell integrity (**B**). Rod-specific deletion of PKM2 alters gene expression (**C**). Loss of PKM2 in the RPE disrupts RPE integrity and affects RPE65-dependent visual cycle function, further impacting retinal health (**D**). Combined Abca4 deficiency and rod-specific PKM2 deletion results in rhodopsin mislocalization, loss of retinal structure and function, and retinal degeneration (**E**). These findings indicate that PKM2 plays an important role in both the RPE and photoreceptors, and that their interdependence is essential for maintaining retinal metabolism, RPE integrity, and photoreceptor survival.

In summary, we examined the roles of PKM isoforms in retinal development, metabolism, and photoreceptor function (Fig. 9). PKM2 is expressed early in retinal development, whereas PKM1 expression occurs later in different retinal cell types. Pan-retinal deletion of PKM2 alters the cone opsin gradient, retinal metabolism, and RPE integrity. Rod-specific deletion of PKM2 alters gene expression. Loss of PKM2 in the RPE disrupts RPE integrity and RPE65-dependent visual cycle function. Combined Abca4 deficiency and rod-specific PKM2 deletion leads to rhodopsin mislocalization, loss of retinal structure and function, and retinal degeneration. These findings indicate that PKM2 plays an important role in retinal metabolism, RPE integrity, and photoreceptor survival and support the interdependence between photoreceptors and the RPE in maintaining retinal homeostasis.

## Materials and methods

### Animals

All animal procedures followed the ethical guidelines outlined in the ARVO Statement for the Use of Animals in Ophthalmic and Vision Research and adhered to the National Institutes of Health Guide for the Care and Use of Laboratory Animals. Experimental protocols were approved by the Institutional Animal Care and Use Committee (IACUC) at the University of Oklahoma Health Sciences Center. Breeding stocks of Pkm2 (Jax #024048), Chx10-Cre (Jax #005105), *Abca4*^*Ko*^ (Jax #023725), and RiboTag (Jax #011029) mice were obtained from The Jackson Laboratory (Bar Harbor, ME). Rhodopsin-Cre (i75Cre) mice were generously provided by Dr. Ching-Kang Jason Chen from Baylor College of Medicine (Houston, TX). Mice were bred and housed in our institutional animal facility under a controlled light environment (12-hour light/dark cycle, 40–60 lux). All animals were tested for the presence of the *rd1* and *rd8* retinal degeneration mutations and confirmed negative. To prevent experimental bias, mice were randomly assigned to groups matched for sex, age, and genetic background. Litters were combined to eliminate potential litter-specific effects.

### Pyruvate kinase activity assay

Pyruvate kinase activity was assessed using a lactate dehydrogenase–coupled assay [17]. Mouse retinal lysates were added to a reaction mixture containing 50 mM Tris-HCl (pH 7.4), 100 mM KCl, 5 mM MgCl₂, 1 mM ADP, 0.5 mM phosphoenolpyruvate, 0.2 mM NADH, and 8 U LDH. Enzyme activity was determined by monitoring the decrease in absorbance at 340 nm due to NADH oxidation. The rate of change in absorbance was used to calculate pyruvate kinase activity.

### Lactate release assay

Lactate release from retinal tissue was measured using an ex vivo incubation method [17]. Isolated retinas were incubated in Krebs–Ringer bicarbonate (KRB) buffer containing 5 mM glucose. After 30 min, the incubation medium was collected, and the lactate concentration was determined using a lactate assay kit (Trinity Biotech) according to the manufacturer’s instructions.

### Aldolase activity

Aldolase activity was measured in a reaction mixture containing 0.1 M potassium acetate buffer (pH 8.0), 0.1–5 mM fructose-1,6-bisphosphate (FBP), 0.2 mM NADH, and the coupling enzymes glycerol phosphate dehydrogenase and triosephosphate isomerase. The reaction was initiated by adding retinal lysate, and activity was determined by monitoring the decrease in absorbance at 340 nm due to NADH oxidation. Relative activity was normalized to protein concentration.

### ATP measurement

ATP levels were determined using the EnzyLight ATP Assay Kit (BioAssay Systems, Hayward, CA) according to the manufacturer’s instructions.

### Generation of rod-specific Pkm2/Ribo-Tag mice

To generate rod-specific Pkm2/Ribo-Tag mice, we initially crossed homozygous *Pkm2* floxed mice with homozygous Ribo-Tag mice. In parallel, a separate breeding was performed using homozygous Ribo-Tag mice and rhodopsin-Cre transgenic mice. Male offspring carrying a single floxed Pkm2 allele, homozygous for the Ribo-Tag, and expressing rhodopsin-Cre were subsequently bred with females homozygous for both *Pkm2* and Ribo-Tag. This breeding scheme produced two experimental groups: Cre-positive mice heterozygous for Pkm2 (*Pkm2*^*Rodhet*^, designated WT) and Cre-positive mice lacking both Pkm2 alleles (*Pkm2*^*Rodko*^, designated KO). All resulting mice were homozygous for the Ribo-Tag allele. Retinal tissues from two-month-old mice were then processed for polyribosome immunoprecipitation, as previously described [29]. Both the input and HA-polyribosome immunoprecipitated RNA fractions were submitted for RNA sequencing (targeting 240 million reads) at MedGenome Inc. (Foster City, CA).

### Statistical analysis

Sample sizes were determined using power calculations as previously described [48]. To minimize bias, animals were assigned to experimental groups based on sex, age, and strain, and littermates were distributed among groups to avoid litter effects. All animals were genotyped, assigned unique identification numbers, and randomly selected so that investigators remained blinded during experimentation and data analysis. All data were analyzed with GraphPad Prism version 7.3. No data points were excluded from the analysis. The selection of statistical tests was based on a specific experimental design. Before analysis, the data sets were tested for normality using the Anderson-Darling, D’Agostino-Pearson, Shapiro-Wilk, and Kolmogorov-Smirnov tests to determine whether they followed a normal or non-normal distribution. For data sets that did not meet normality criteria, non-parametric tests were used to compare groups, specifically multiple Mann-Whitney U tests. To control multiple comparisons and limit the false discovery rate, the two-stage Benjamini-Krieger-Yekutieli procedure was applied with a Q value set at 1%. For normally distributed data, comparisons between two groups were performed using parametric tests with Welch’s correction to account for unequal variances. The significance of differences was determined based on p-values. One-way analysis of variance (ANOVA) was used to identify differences among three or more independent groups, while two-way ANOVA was applied to evaluate the effects of two categorical factors on a continuous outcome variable.

### Other methods

Translation Ribosome Affinity Purification (TRAP) was used to isolate actively translating mRNAs from retinal cells, followed by RNA extraction and analysis, as described previously [15, 17]. Electroretinography (ERG) was performed under scotopic and photopic conditions to assess retinal function [16]. Tissue histology was carried out on fixed retinal sections stained with hematoxylin and eosin to evaluate morphology [16].

Immunohistochemical staining was conducted on retinal sections to assess protein localization. qRT-PCR was performed using gene-specific primers (Table S3; Fig. S7) and normalized to housekeeping genes. Western blotting was performed using protein lysates separated by SDS–PAGE, transferred to membranes, and probed with specific antibodies. Antibodies used in this study are listed in Table S4.

Steady-state metabolite profiling was performed using gas chromatography–mass spectrometry (GC–MS) as previously described [27]. Metabolites were extracted, derivatized, and analyzed by GC–MS, and the data were normalized to the internal standard ribitol and tissue weight.

## Supplementary information


Supplemental Data File
Uncropped Western blot images


## Data Availability

All data generated or analyzed during this study are included in this published article. The TRAP data reported here (*Pkm2*^*-/-*^ and Abca4^*-/-*^) have been deposited in the Gene Expression Omnibus with accession numbers GSE270134 and GSE298433. The quantitative proteomics data have been deposited in Zenodo under accession number 10.5281/zenodo.19134323.
