# Supplementary material for: Interdependent roles of PKM2 in photoreceptors and RPE: implications for retinal degeneration
Source: Cell Death Dis. 2026 Jun 19;17(1):761. doi: 10.1038/s41419-026-08997-3 (PMC13527088; doi:10.1038/s41419-026-08997-3)
Supplement: Supplementary file 1 [file 41419_2026_8997_MOESM1_ESM.pdf]

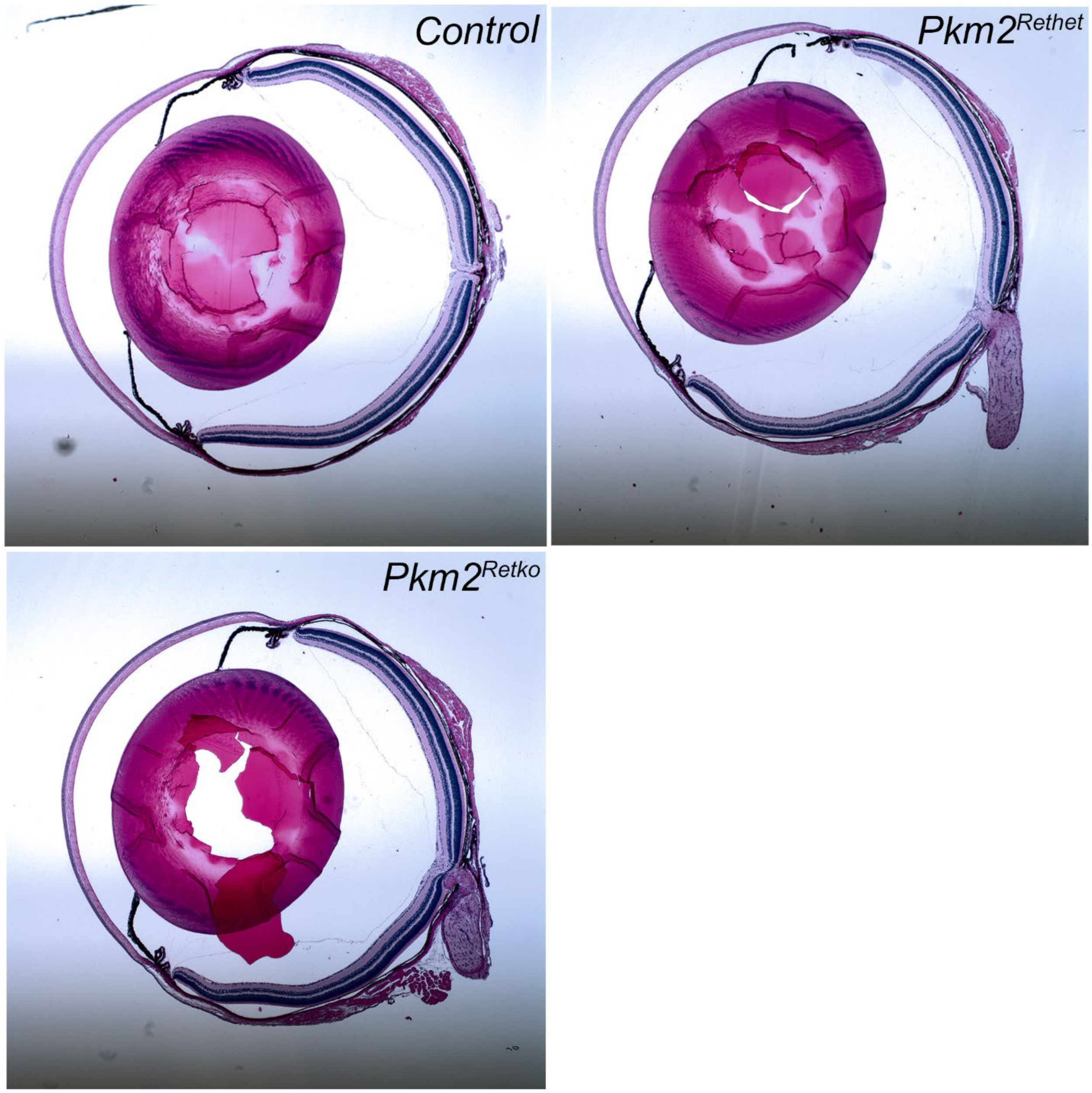

Figure S1. Retinal histology of retina-specific PKM2 KO mice and assessment of rod outer segment integrity. Retinal histology was performed on one-month-old control (*Pkm2*$^{flox/flox}$), *Pkm2*^{Rethet}, and *Pkm*2^{Retko} mice with hematoxylin and eosin staining.

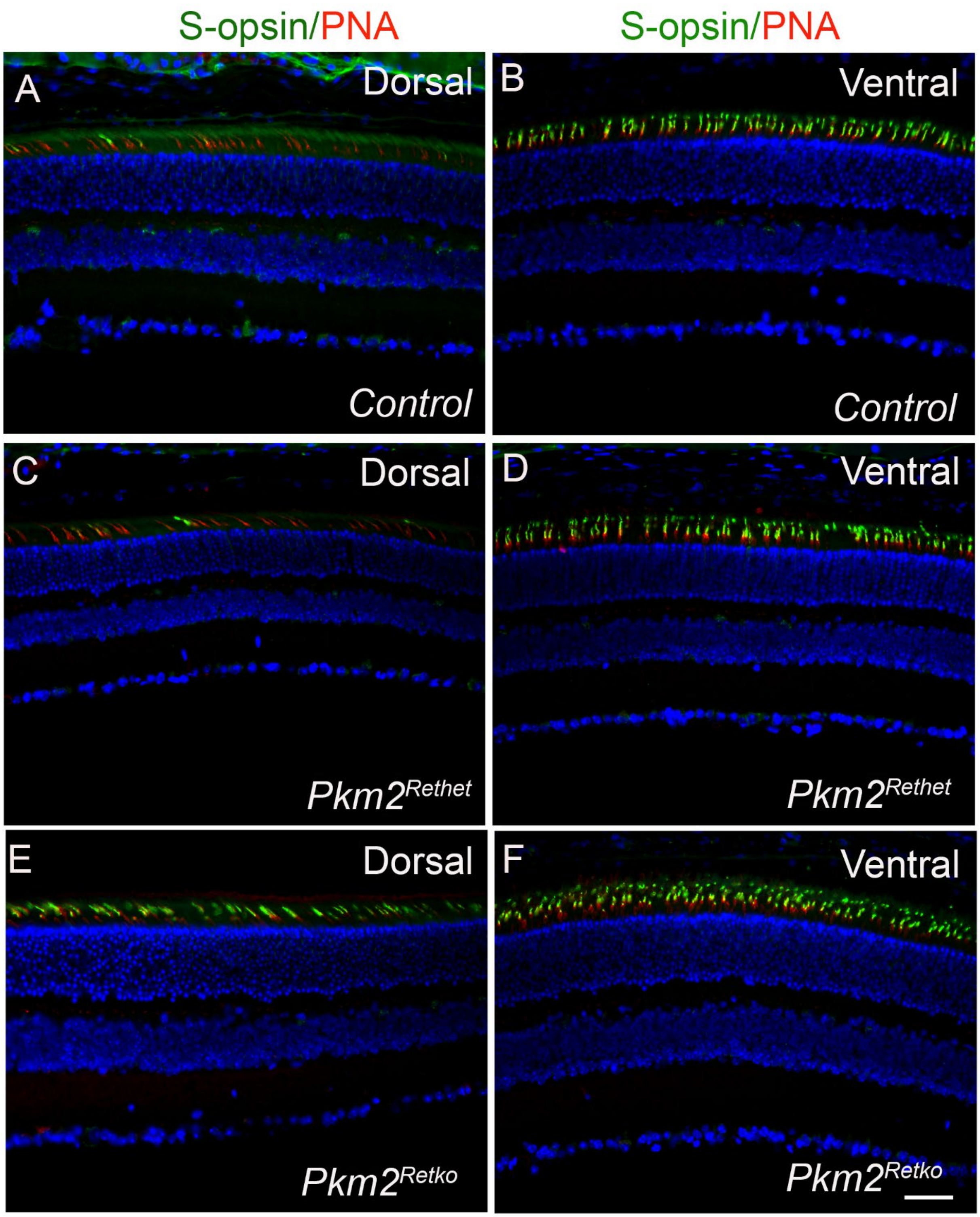

Figure S2. Altered cone-opsin gradient in *Pkm2*^{Retko} mice. Expression of S-opsin (S-opsin positive cones) in dorsal and ventral regions of the retina from one-month-old control (A, B), Pkm2^{Rethet,} (C, D) and *Pkm2*^{Retko} (E, F) mice. Retina sections were stained with S-opsin and PNA.

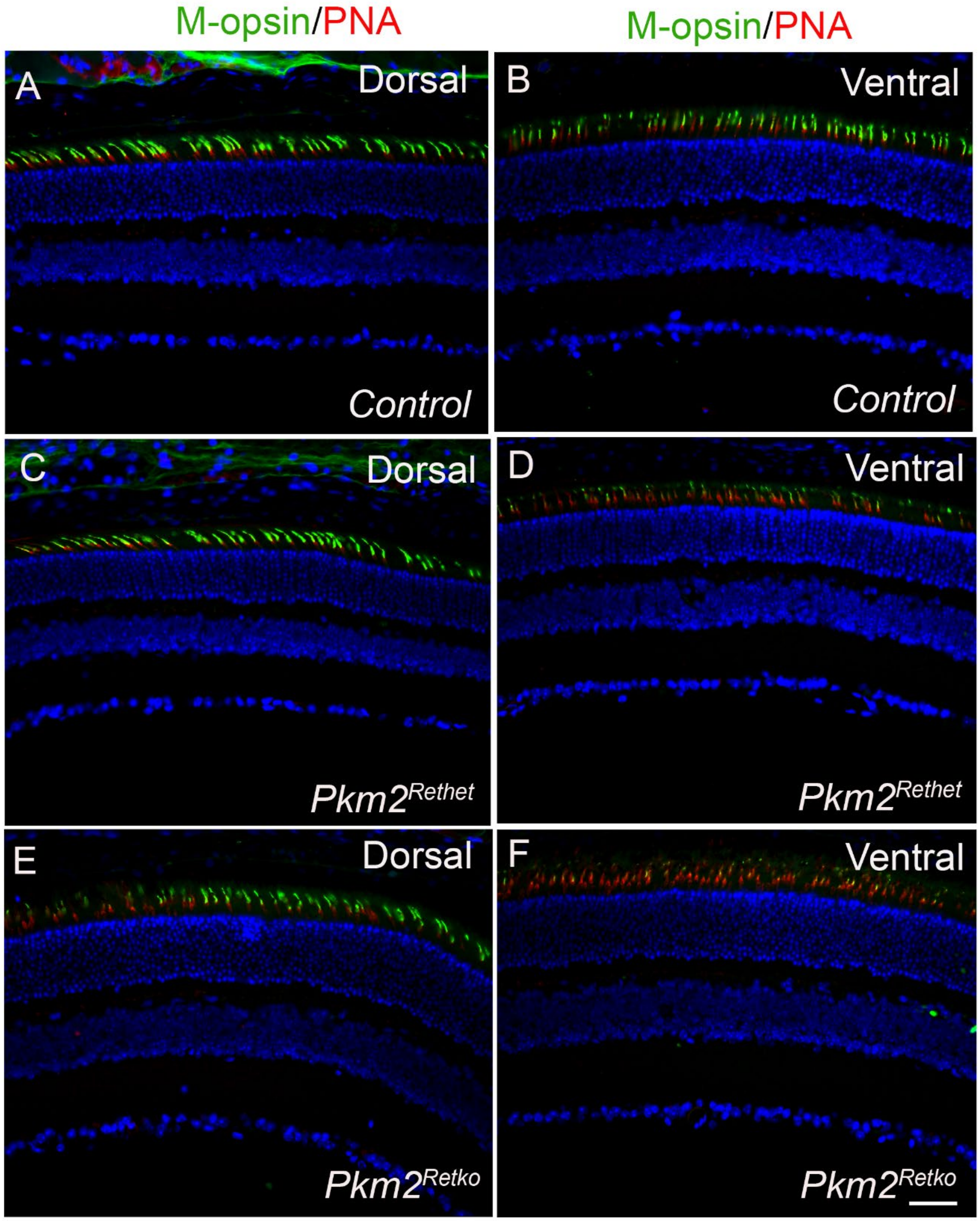

Figure S3. Cone-opsin gradient in *Pkm2^{Retko}* mice. Expression of M-opsin (M-opsin positive cones) in dorsal and ventral regions of the retina from one-month-old control (A, B), Pkm2^{Rethet} (C, D) and *Pkm2^{Retko}* (E, F) mice. Retina sections were stained with M-opsin and PNA.

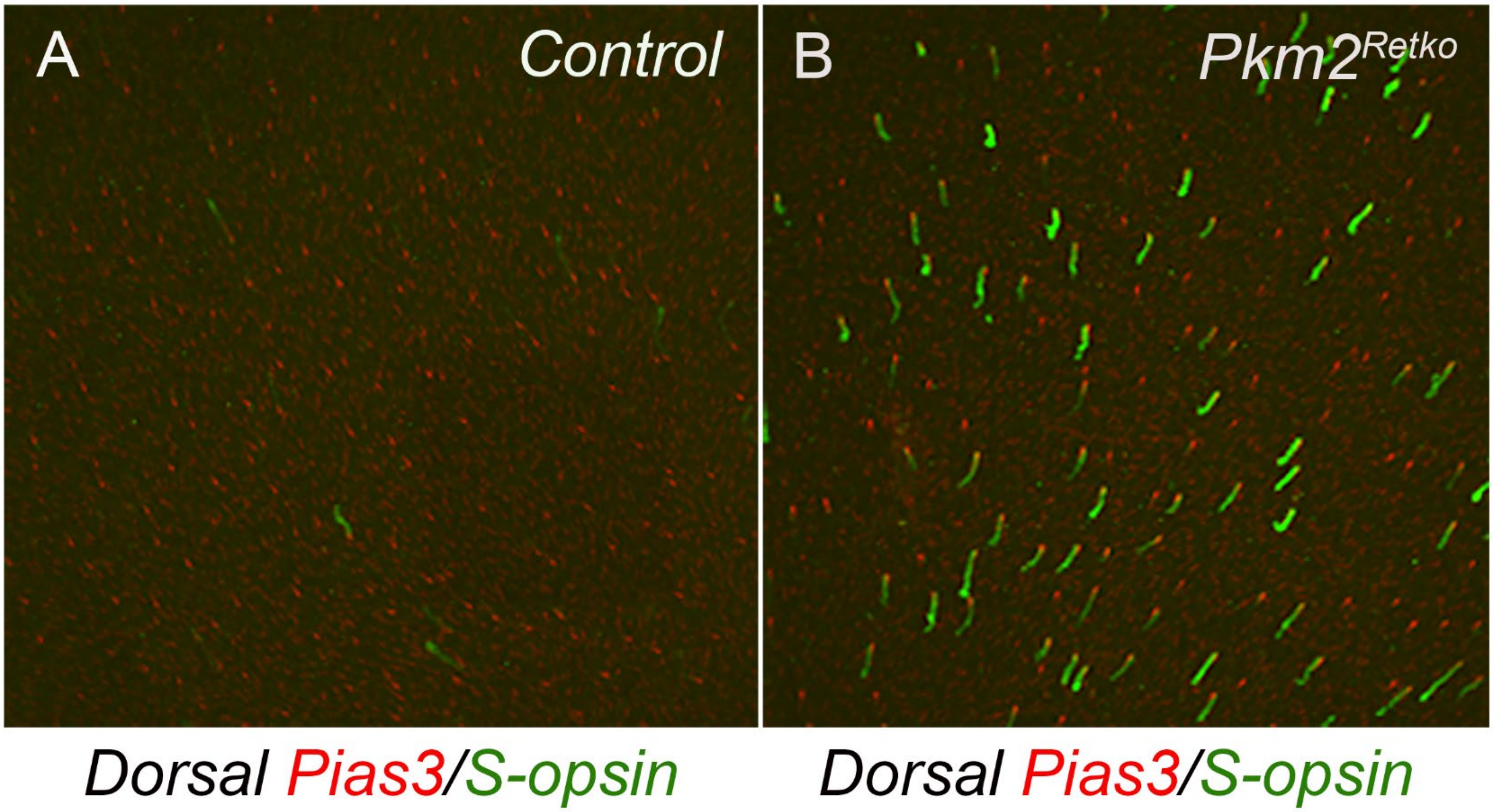

Figure S4. Higher magnification images of PIAS3 and S-opsin staining from Figure 3I. Retinal flat mounts from control (A) and *Pkm2*^{Retko} (B) mice were immunostained with antibodies against pias3 and S-opsin and examined in the dorsal region of the retina to assess their expression patterns.

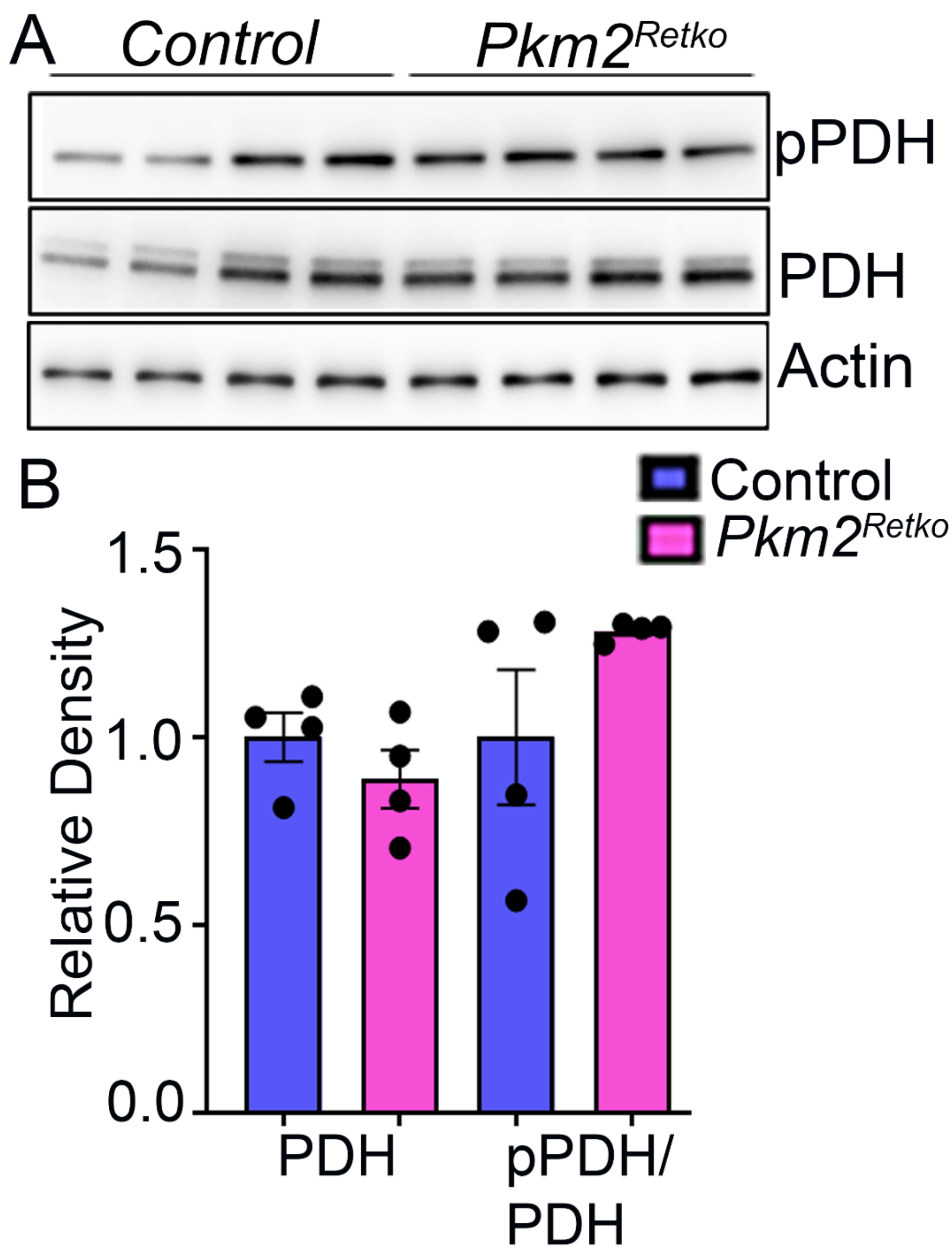

Figure S5. Effect of PKM2 loss on PDH expression and phosphorylation in the retina. Retinal lysates from control and *Pkm2*^{Retko} mice were immunoblotted with antibodies against pPDH, PDH, and actin (A). Densitometric analysis of the pPDH/PDH ratio is shown in (B). Data represent mean ± *SEM (n = 4).*

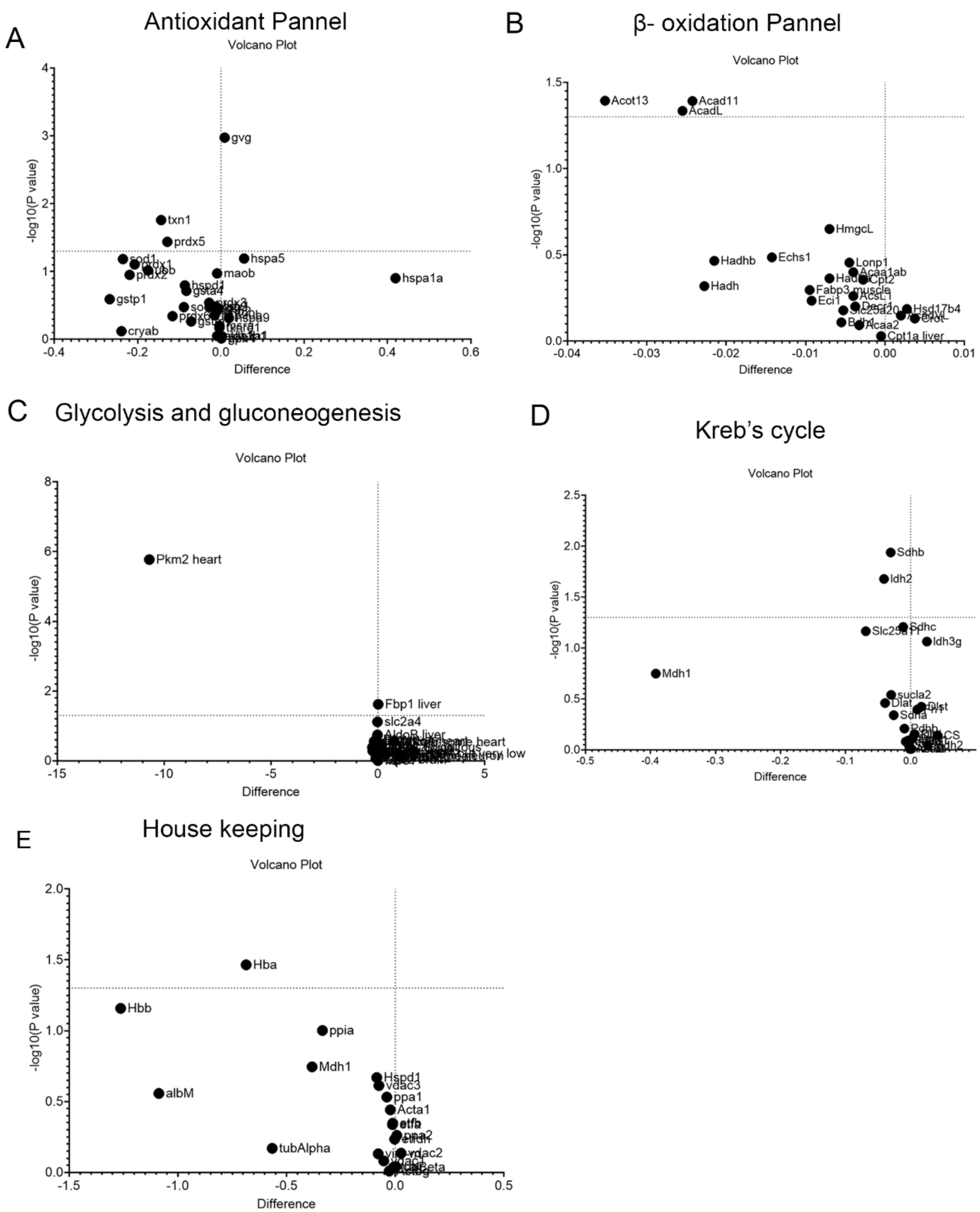

Figure S6. Quantitative proteomic analysis of control and *Pkm2*^{Retko} mouse retina. Retinal proteins were extracted, digested with trypsin, and analyzed by liquid chromatography–tandem mass spectrometry (LC-MS/MS). Targeted quantitative proteomic analysis was then used to measure antioxidant/stress response proteins (A), β-oxidation enzymes (B), glycolytic and gluconeogenic enzymes (C), Krebs cycle enzymes (D), and housekeeping proteins (E). Volcano plots of protein levels in the retina of control and *Pkm2*^{Retko} mice. Each point represents an individual protein quantified from four biological replicates per group (mean ± *SEM*).

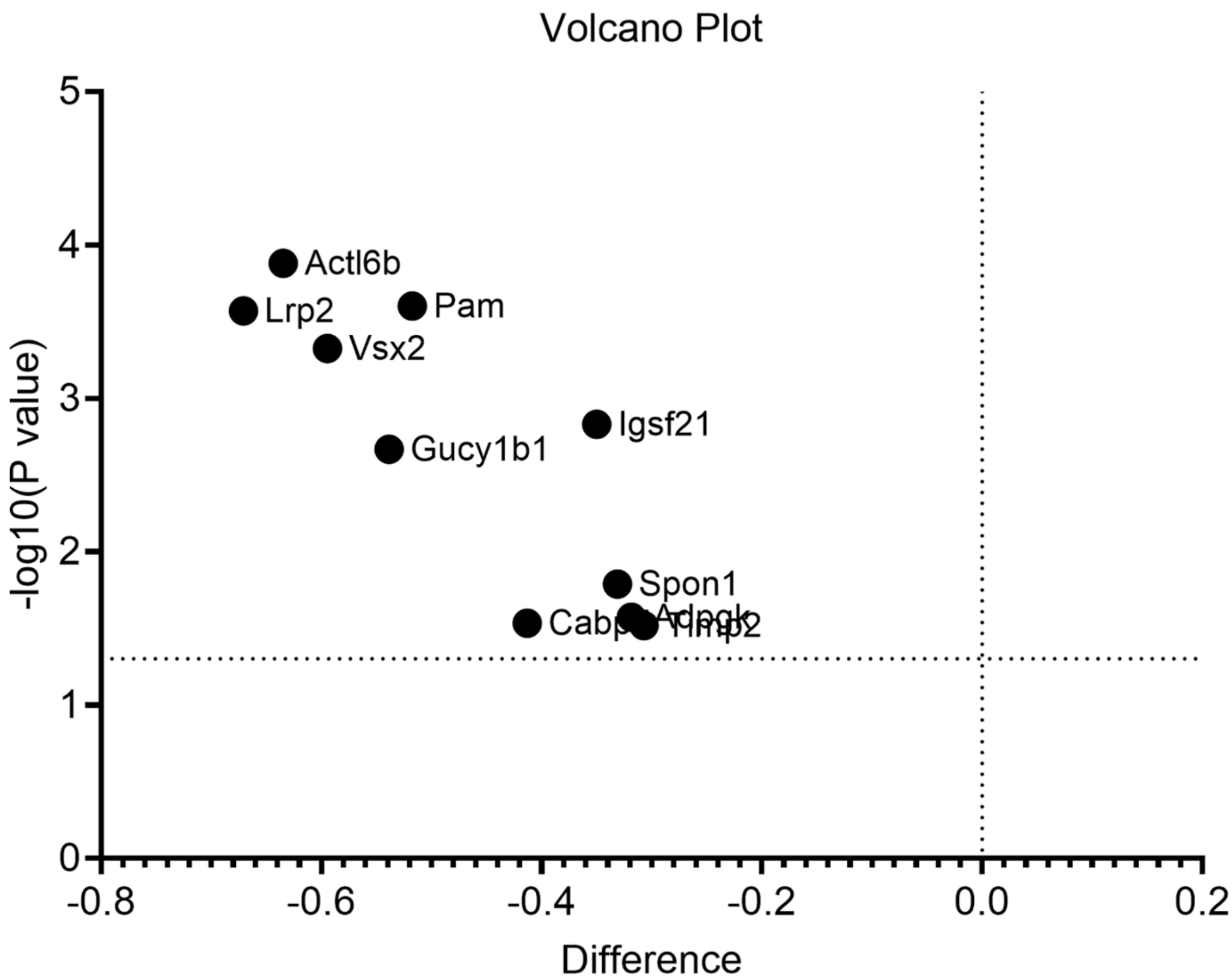

Figure S7. Validation of Selected Candidate Genes by Quantitative Real-Time PCR. Equal amounts of retinal mRNA from three independent two-month-old *control* and *Pkm2*^{Rodko} mice were used for qRT-PCR and normalized to *Rpl37* levels. The mRNA levels were averaged for *Actl6b, Adpgk, Lrp2, Pam, Vsx2, Gucy1b1, Igsf21, Spon1, Timp2,* and *Cabp5.* The data are presented as mean ± *SEM (n=3).*

Real-time PCR primers are used to measure gene expression.		
**Gene**	**Forward Primer**	**Reverse Primer**
Adpgk	CCCAGATAAGCCAGTGGTGG	GCATCACCAAGGCCTACAGT
Actl6b	TGACGGAGCTGATGTTCGAG	GACGATGCCTTGCTGTAGGA
Lrp2	TGACTGTGTCGACGGTTCTG	CGGTGTCTGTGCAAGGTTTG
Pam	TGTGTTTGCCTACCGAGTCC	GGTAAAAAGCCTGTGGCAGC
Vsx2	GCGTCATGGCTGAGTATGGT	TTCGGGTTTAGAAAAGCGCC

Gucy1b1	TTTGGGAGAACAGTTCCGGG	TCCTCCAAGGCTCTCAGTGT
Igsf21	GTATCTACGACCGAGCCACG	AGTGTGAAGTTCTGGGCCTG
Spon1	GCCAGAGTCGTCATCGAGAG	CGTCCACGTTGTCAGGTACA
Timp2	ACTCTCAAGCTTCCCAAGCC	CTGATGCGACAGAGAGCCAA
Cabp5	AATCAGGAAGCTGGGTTGCC	GGCAGGACCCATTGGAAACT
Rpl37	CGGGACTGGTCGGATGAG	TCACGGAATCCATGTCTGAATC

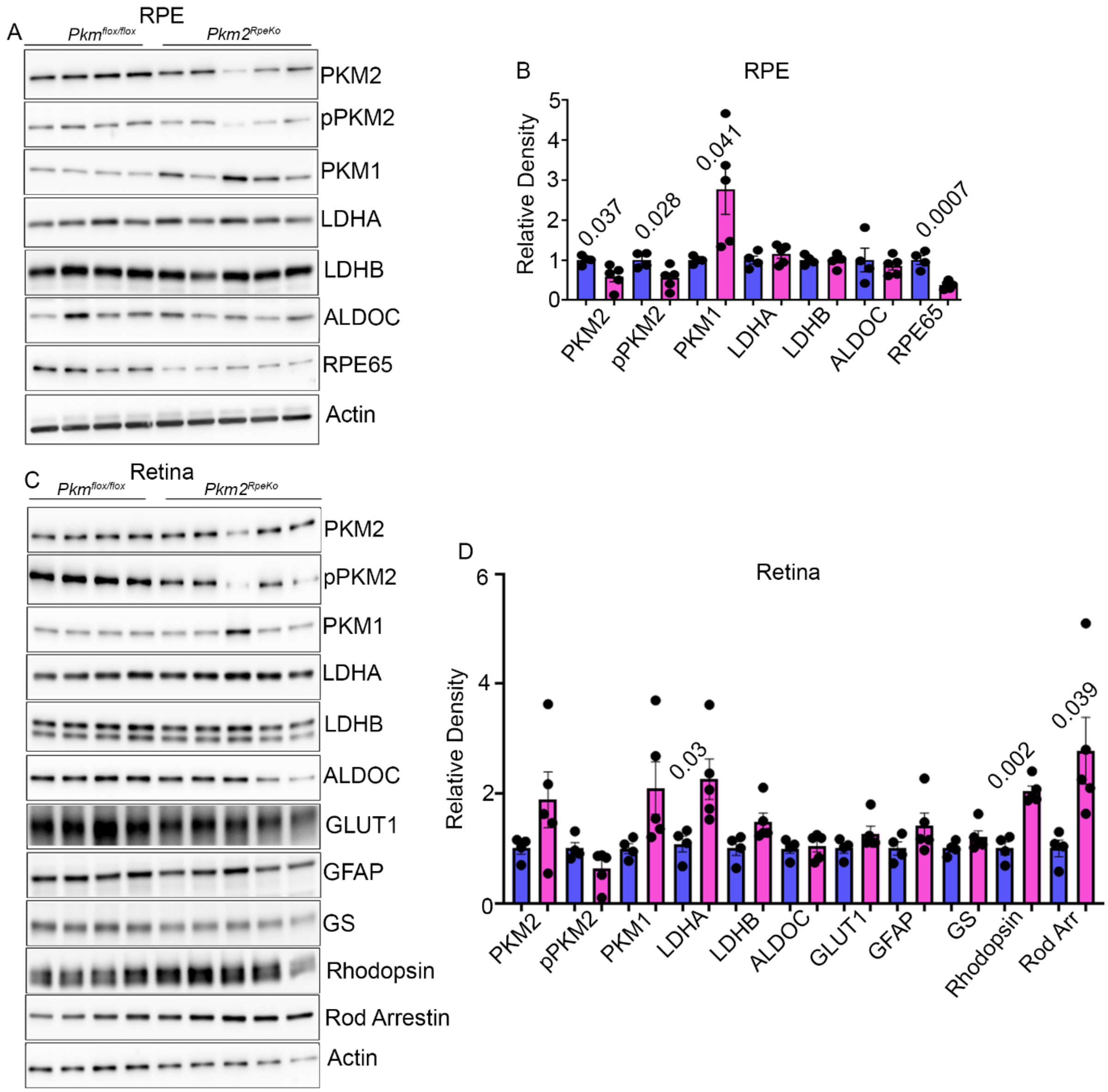

Figure S8. Characterization of RPE-specific PKM2 KO (*Pkm2^Rpeko^*) mice and its effect on RPE and photoreceptor cells. RPE (A, B) and retina (C, D) were isolated from one-month-old Control (*$Pkm2^{flox/flox}$*) and *$Pkm2^{Rpeko}$* mice and immunoblotted with PKM2, pPKM2, PKM1, LDHA, LDHB, ALDOC, GLUT1, GFAP, GS, rhodopsin, RPE65, rod arrestin, and actin antibodies. Immunoblotted proteins were normalized to actin (B, D). Data are mean ± *SEM (n= $Pkm2^{flox/flox}$ 4; $Pkm2^{Rpeko}$ 5).*

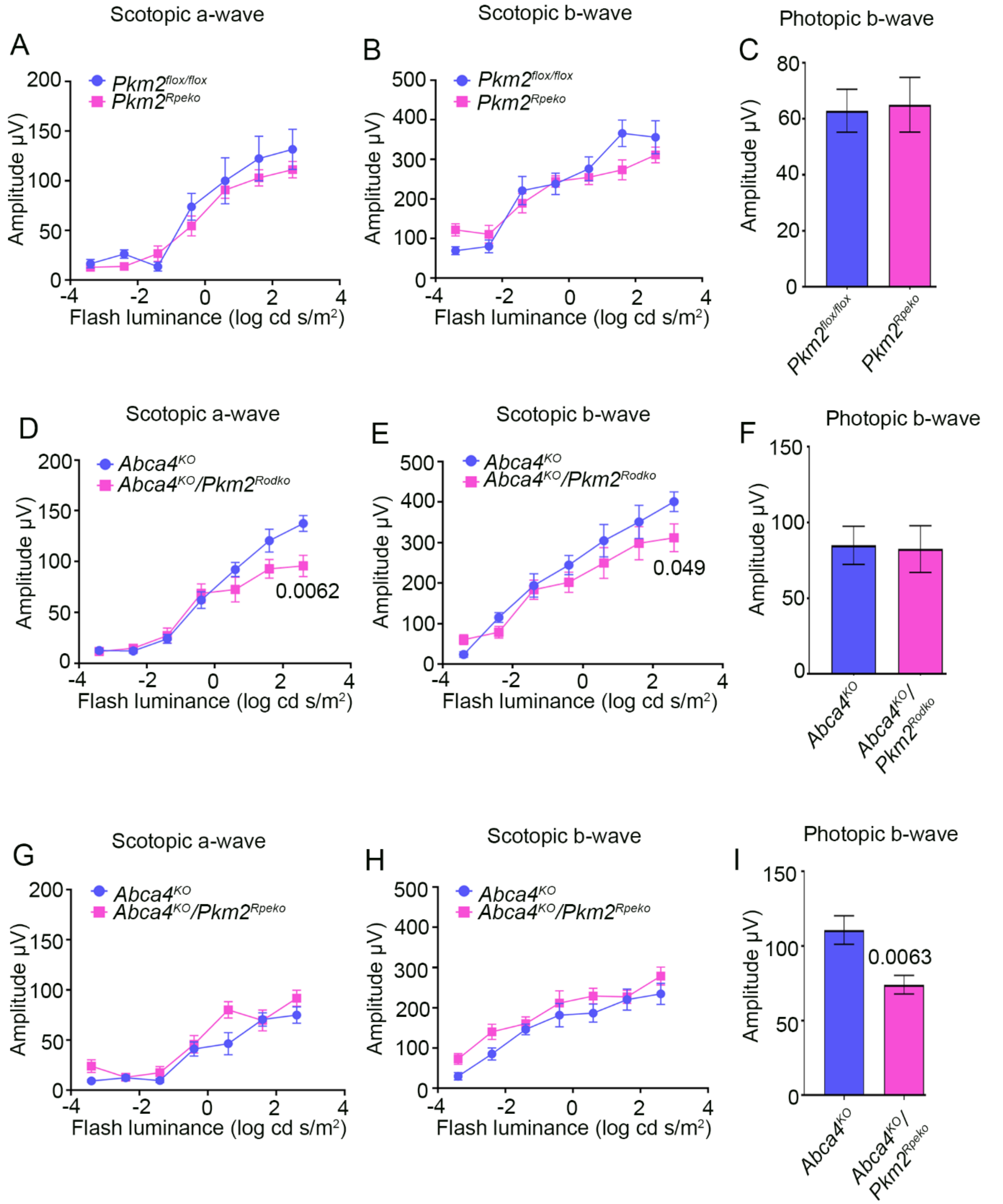

Figure S9. Functional Characterization of *Pkm2^Rpeko^*, *Abca4^Ko^/Pkm2^Rodko^*, and *Abca4^Ko^/Pkm2^Rpeko^* mice. Scotopic a-wave (A, D, G), scotopic b-wave (B, E, H), and photopic b-wave (C, F, I) amplitudes in 2.5-month-old *$Pkm2^{Rpeko}$* (A-C), 3-month-old *$Abca4^{Ko}/Pkm2^{Rodko}$* (D-F), and 5-month-old *$Abca4^{Ko}/Pkm2^{Rpeko}$ mice* (G-I), along with littermate controls. Data are mean $\pm$ *SEM (n=8).*

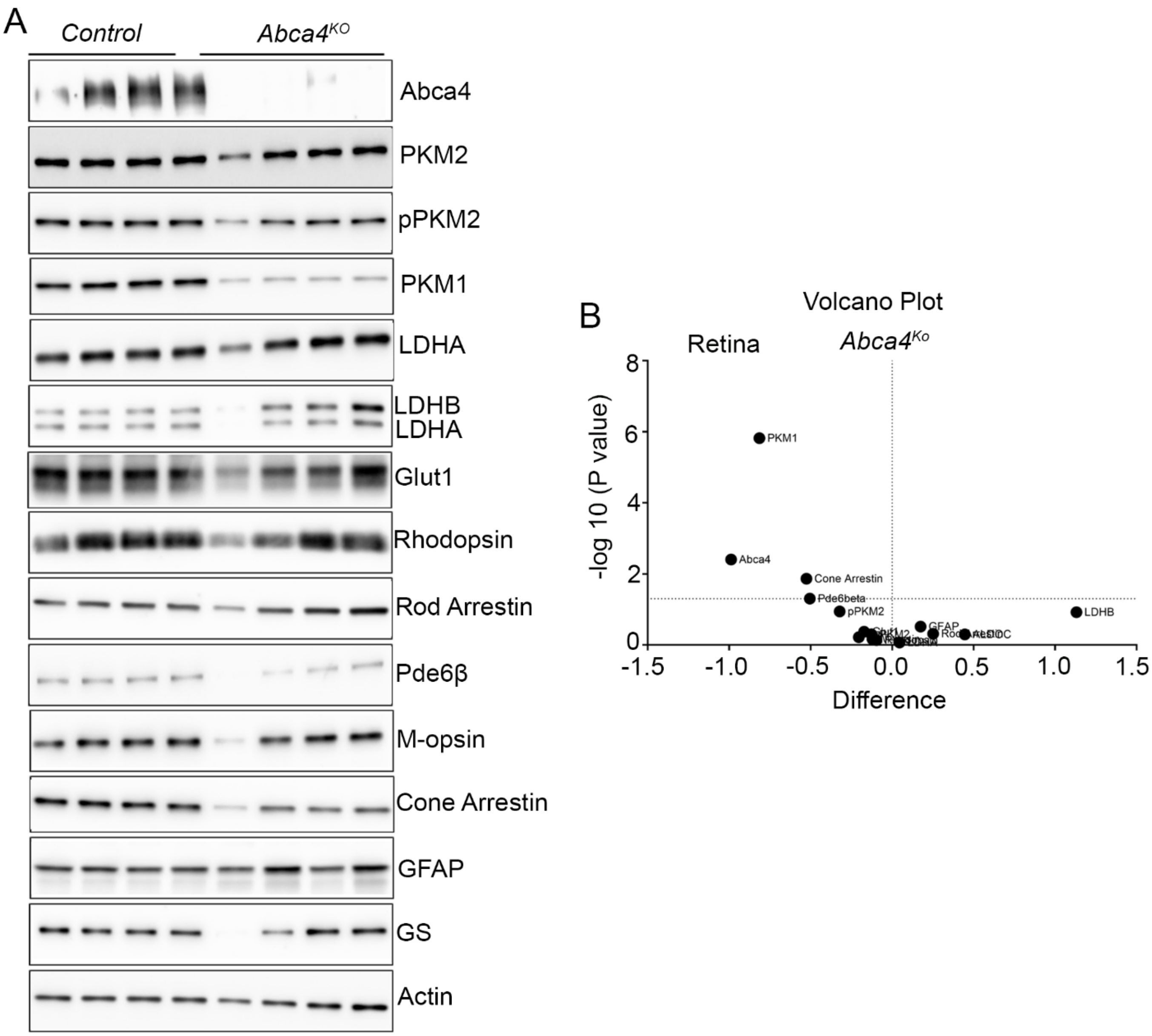

Figure S10. Characterization of *Abca4^{Ko}* mice. Retinal proteins from *Abca4*^{Ko} and its littermate control mice were immunoblotted with Abca4, PKM2, pPKM2, PKM1, LDHA, LDHB, Glut1, Rhodopsin, rod arrestin, Pde6β, M-opsin, cone-arrestin, GFAP, GS, and actin antibodies (A). Volcano plots of protein levels in retina from control and *Abca4^{Ko}* mice (B). Each point represents an individual protein quantified from four biological replicates per group (mean ± *SEM*).

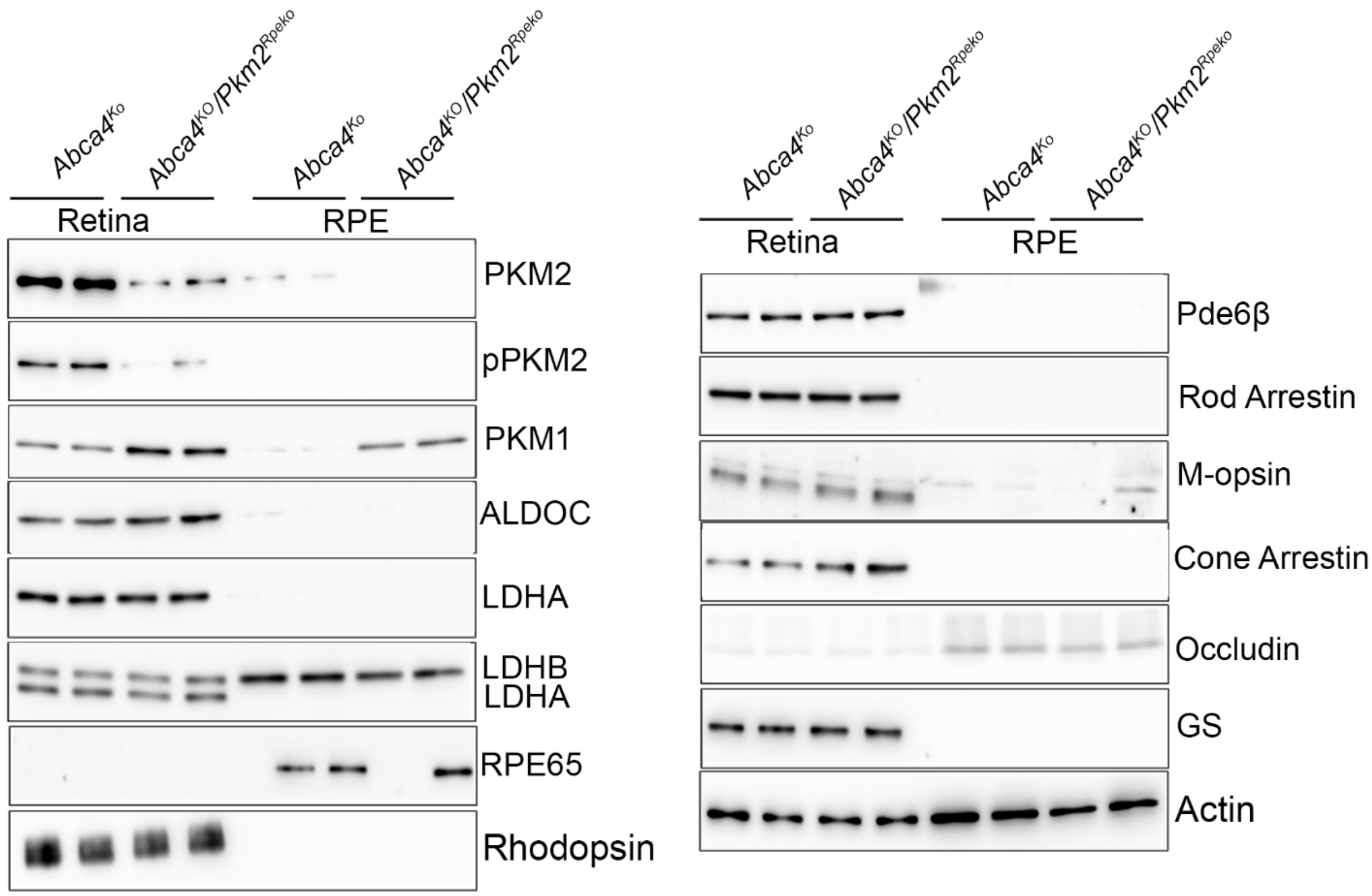

Figure S11. Characterization of *Abca4^{Ko}* and *Abca4^{Ko}/Pkm2^{Rpeko}* mice and its effect on RPE and retina. RPE and retina were isolated from two-month-old *Abca4^{Ko}* and *Abca4^{Ko}/Pkm2^{Rpeko}* mice and immunoblotted with antibodies against PKM2, pPKM2, PKM1, ALDOC, LDHA, LDHB, RPE65, rhodopsin, Pde6β, rod arrestin, M-opsin, cone arrestin, occludin, GS, and actin.

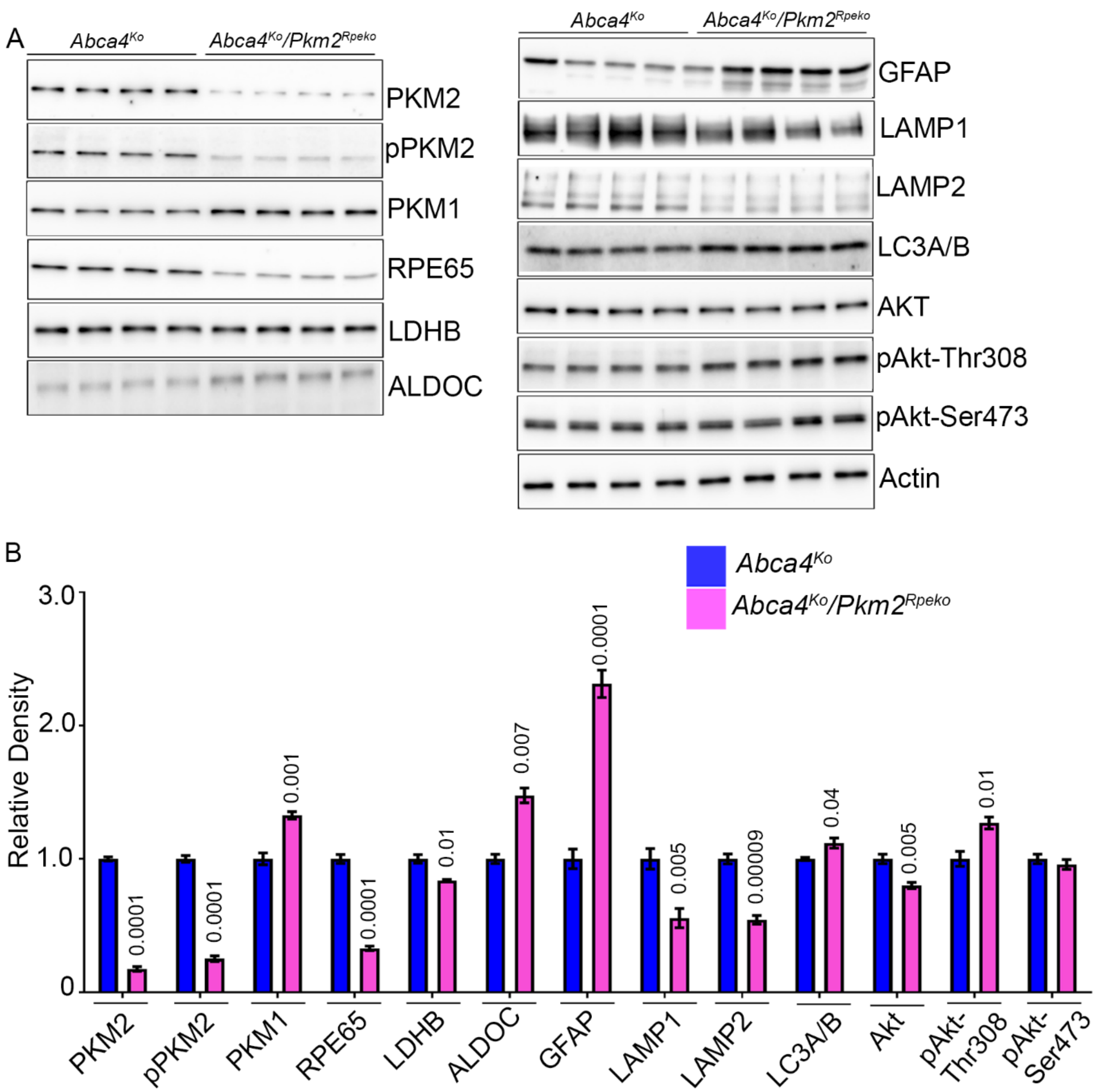

Figure S12. Characterization of three-month-old *Abca4^Ko^* and *Abca4^Ko^/Pkm2^Rpeko^* mice and effects on RPE. RPE lysates from three-month-old *Abca4^{Ko}* and *Abca4^{Ko}/Pkm2^{Rpeko}* mice were immunoblotted with antibodies against PKM2, pPKM2, PKM1, RPE65, LDHB, ALDOC, GFAP, LAMP1, LAMP2, LC3A/B, Akt, pAkt-Thr308, pAkt-Ser473, and actin (A). Densitometric values of each protein were normalized to actin (B). Data are mean ± *SEM (n =4).*

Table S1. Targeted metabolites analyzed in retinal samples

Glycolysis/Sugar Metabolism	TCA Cyle	Amino Acids	Lipid Metabolism	Other Metabolites
Glucose	Citrate	Ala	Palmitate	Creatinine
Fructose	Succinate	Asp	Stearate	Urea
Galactose	Fumarate	Ser	3-Hydroxybutyrate	Taurine
Mannose	Malate	Thr		Phosphoric acid
G6P		Gly		*Myo*-inositol
Pyruvate		Pro		Oxoproline
Lactate		Glu		Aminomalonic acid
		Leu		Ethanimidic acid
		GABA		Toluic acid
		2-Aminoadipic acid		

Table S2

RNA Sequencing Data on TRAP (control and Abca4 KO mice)

			Contorl -IP	Control-IP	Control-IP	Abca4-IP	Abca4-IP	Abca4-IP
name	gene_nam	PAdj	X03.bam.c	X03.bam.c	X03.bam.c	X03.bam.al	X03.bam.al	X03.bam.al
ENSMUSG(	Polr3d	5.56E-05	225	187	165	316	338	356
ENSMUSG(	Scn4a	0.0249	1278	1018	1064	732	833	812
ENSMUSG(	Tcea3	0.000757	140	116	153	208	280	454
ENSMUSG(	Slc1a5	4.29E-07	137	142	176	266	370	426
ENSMUSG(	Wwox	0.022344	1010	1095	1084	553	821	758
ENSMUSG(	Ccn4	0.000587	1015	1207	1360	716	814	679
ENSMUSG(	Eps8l1	7.69E-08	582	971	933	50	44	0
ENSMUSG(	Cdcp3	0.000855	1901	2385	2326	1296	1591	1313
ENSMUSG(	Scube2	0.001644	139	123	115	66	85	65
ENSMUSG(	Sirt2	2.38E-07	1860	1660	1574	3222	2738	2960
ENSMUSG(	Cltrn	0.001787	126	102	140	191	199	288
ENSMUSG(	Camk1g	5.18E-06	204	164	163	86	99	84
ENSMUSG(	Isyna1	0.031554	609	792	741	911	1009	1360
ENSMUSG(	Arfgef3	0.000338	205	220	202	60	116	120
ENSMUSG(	Tcn2	5.01E-05	686	848	782	1405	1120	1438
ENSMUSG(	Myo1g	0.004143	82	89	63	27	53	23
ENSMUSG(	Rab37	0.008854	145	186	184	205	397	1434
ENSMUSG(	Slc9a3r1	0.014632	152	165	190	239	279	609
ENSMUSG(	Lpcat1	0.033483	4461	4422	4080	6992	5424	5506
ENSMUSG(	Enox1	0.004073	850	1092	981	428	226	643
ENSMUSG(	Rb1	0.004887	673	678	675	854	1172	1039
ENSMUSG(	Gsc2	0.02193	78	108	148	173	188	372
ENSMUSG(	Ncbp2	0.012369	1121	1066	944	1491	1390	1498
ENSMUSG(	Mylk	0.045527	2452	1995	1836	2622	2909	3393
ENSMUSG(	Krt18	9.63E-08	1960	1673	1765	3808	3434	2868
ENSMUSG(	Poc1a	0.024187	136	112	116	89	76	66
ENSMUSG(	Tnfrsf12a	0.022139	67	71	94	129	124	115
ENSMUSG(	Rab3il1	1.03E-05	115	124	97	216	189	222
ENSMUSG(	Ccdc51	0.020991	557	651	602	359	478	400
ENSMUSG(	Gdap1	0.022139	470	502	537	268	376	365
ENSMUSG(	Ly75	0.000839	67	86	68	23	44	37
ENSMUSG(	Tyro3	1.21E-05	610	452	452	172	294	209
ENSMUSG(	Hdc	0.008685	675	881	842	550	477	278
ENSMUSG(	Tgm3	0.044324	5	10	3	8	11	149
ENSMUSG(	Fam210b	1.24E-09	541	469	540	912	1148	1202
ENSMUSG(	Aurka	0.000987	52	36	60	79	111	108
ENSMUSG(	Dclk1	0.009431	741	845	841	555	605	552
ENSMUSG(	Sass6	0.042629	75	61	70	82	130	130
ENSMUSG(	Extl2	0.026642	1728	1691	1594	1102	1295	1274
ENSMUSG(	Bcar3	4.94E-11	586	534	526	1075	1102	1014
ENSMUSG(	Abca4	0.01936	61017	53193	53533	195	365	13156
ENSMUSG(	Tra2a	0.003564	1373	1233	1249	1810	1744	1943
ENSMUSG(	Ercc2	0.019387	905	1003	892	742	627	529
ENSMUSG(	Pop4	6.77E-05	361	404	381	139	219	228

ENSMUSG(	Pcsk6	6.87E-54	6	6	4	255	170	298
ENSMUSG(	Synm	0.001486	1317	1365	1385	656	708	1032
ENSMUSG(	Gdpd3	1.29E-09	1	1	1	58	47	12
ENSMUSG(	Smarca1	0.049324	40	33	22	13	19	14
ENSMUSG(	Adprhl1	0.004439	62	68	56	35	29	35
ENSMUSG(	Tbc1d9	3.00E-05	1256	1145	1049	763	553	484
ENSMUSG(	Btg4	0.00692	20	20	18	5	8	7
ENSMUSG(	Mns1	1.21E-05	437	338	300	151	161	208
ENSMUSG(	Nrg4	0.023227	39	36	53	28	16	20
ENSMUSG(	Aph1b	0.034471	293	324	334	198	214	251
ENSMUSG(	Nmnat3	2.66E-05	76	87	87	33	41	41
ENSMUSG(	Mrpl3	0.021293	357	416	368	484	576	568
ENSMUSG(	Ip6k2	0.048356	2519	3575	2718	7688	3947	3700
ENSMUSG(	A930018P2	0.041649	59	56	60	30	39	35
ENSMUSG(	Agl	5.01E-05	457	489	473	665	1075	980
ENSMUSG(	Upb1	0.010287	24	32	49	14	13	18
ENSMUSG(	Piwil2	0.000209	70	76	69	19	38	39
ENSMUSG(	Trim36	0.041641	685	675	665	806	969	1010
ENSMUSG(	Meak7	0.009504	1140	1206	1184	852	855	861
ENSMUSG(	Rhbdd3	0.041095	160	179	183	225	254	260
ENSMUSG(	Uaca	5.50E-08	67	51	77	154	716	533
ENSMUSG(	Gpsm3	0.002807	767	754	790	1030	1124	1407
ENSMUSG(	Sgcg	2.25E-123	1	0	0	689	1080	738
ENSMUSG(	Grhpr	0.028357	463	444	473	335	357	307
ENSMUSG(	Armh4	1.50E-08	19	14	20	1483	43	1596
ENSMUSG(	Clic4	0.016876	355	397	408	503	575	565
ENSMUSG(	Prc1	3.61E-26	21	24	18	113	117	125
ENSMUSG(	Il18	1.93E-16	19	21	14	98	154	87
ENSMUSG(	Nt5dc1	0.044401	211	275	269	137	178	186
ENSMUSG(	Arhgap29	3.85E-06	105	114	156	223	299	279
ENSMUSG(	Apobec2	0.021533	141	111	122	95	48	37
ENSMUSG(	Chrnb1	0.021242	233	253	276	306	447	432
ENSMUSG(	Zfp236	3.71E-05	1486	1454	1506	590	654	1004
ENSMUSG(	Slc7a1	0.000208	484	604	607	260	350	353
ENSMUSG(	Pnpla3	1.72E-05	49	51	38	260	78	239
ENSMUSG(	Haus1	0.001435	187	184	189	114	92	130
ENSMUSG(	Wdr11	0.024306	1016	1061	960	519	659	818
ENSMUSG(	Jph1	0.021888	58	57	53	35	36	23
ENSMUSG(	Otud1	0.015795	278	262	295	152	220	169
ENSMUSG(	Acp1	0.029744	767	701	755	381	608	470
ENSMUSG(	Zbtb7c	0.013442	48	24	6	3	6	6
ENSMUSG(	Zc2hc1c	0.042012	206	294	268	342	374	422
ENSMUSG(	Tmem229b	0.000592	3514	3494	3295	4686	5124	6677
ENSMUSG(	Cfap58	0.000103	4	3	3	8	48	138
ENSMUSG(	Lrrc8e	0.015124	18	12	15	38	43	22
ENSMUSG(	Cd300lf	0.046609	15	20	17	25	32	84
ENSMUSG(	Slc35e4	0.027301	967	1040	1031	1398	1325	1349

ENSMUSG(	Agtr1a	0.026642	16	14	9	46	33	18
ENSMUSG(	Fam241a	0.000154	34	42	69	14	18	18
ENSMUSG(	Gm11868	7.52E-37	3	5	3	86	86	76
ENSMUSG(	Slc29a4	0.016902	35	70	71	60	197	492
ENSMUSG(	Zgrf1	1.74E-05	130	145	121	30	46	73
ENSMUSG(	Dock8	0.024187	230	263	225	354	366	309
ENSMUSG(	Gm9869	1.67E-06	6	25	19	58	184	107
ENSMUSG(	Slc6a2	0.000429	90	38	25	8	11	14
ENSMUSG(	Tescl	2.89E-11	2	0	0	14	25	24
ENSMUSG(	Dnah9	0.038355	412	436	385	260	209	336
ENSMUSG(	Txnl4a	0.009668	356	328	317	586	456	459
ENSMUSG(	Ankrd29	0.037617	139	77	49	22	42	45
ENSMUSG(	Tma7-ps	1.14E-09	1246	1095	1146	3988	2408	2474
ENSMUSG(	Rad51b	0.014288	39	61	48	24	26	26
ENSMUSG(	Ntrk3	0.047325	225	231	248	139	197	131
ENSMUSG(	Rps3a3	0.040527	36	28	28	599	39	33
ENSMUSG(	Tmem26	0.00274	108	108	79	63	53	41
ENSMUSG(	Dcc	3.22E-06	108	91	136	38	39	57
ENSMUSG(	H4c9	0.045278	63	59	64	224	97	84
ENSMUSG(	Ext1	0.007178	1541	1447	1379	1042	1087	824
ENSMUSG(	Zfp27	0.000165	294	340	303	159	191	99
ENSMUSG(	Fabp4	0.039066	53	78	70	30	42	43
ENSMUSG(	Gm12666	6.91E-14	0	7	7	37	41	45
ENSMUSG(	E230001N(	0.004143	1	0	2	57	7	5
ENSMUSG(	Gm10167	1.45E-09	31	39	37	158	162	85
ENSMUSG(	Capza1-ps1	1.01E-06	530	616	548	1065	958	924
ENSMUSG(	Selenbp1	0.001836	146	137	183	1112	333	264
ENSMUSG(	Gm10259	0.027989	1192	1190	1151	1382	1789	1824
ENSMUSG(	Ssbp4	0.005828	2989	3031	3478	4164	4420	5071
ENSMUSG(	Prok1	0.049324	61	110	103	116	179	200
ENSMUSG(	Capza1	0.001614	1513	1543	1481	1037	895	1116
ENSMUSG(	Ces1a	0.00013	0	0	0	0	67	80
ENSMUSG(	Zbtb3	0.004439	72	82	74	116	126	131
ENSMUSG(	Gm5460	0.001095	138	191	177	96	91	104
ENSMUSG(	Srp54a	0.038523	1860	1977	1480	1255	1367	1112
ENSMUSG(	Iqck	0.000702	57	42	49	168	94	89
ENSMUSG(	Ceacam1	0.003405	27	34	20	4	13	10
ENSMUSG(	Rtraf-ps	0.002469	21	25	24	128	28	114
ENSMUSG(	Ccdc162	0.034471	27	21	11	5	4	12
ENSMUSG(	Gm15610	4.13E-41	8	8	13	898	371	425
ENSMUSG(	Mrpl48-ps	0.000557	31	49	35	92	71	76
ENSMUSG(	H2aw	3.46E-08	463	446	598	1037	951	1315
ENSMUSG(	Cfap77	0.007046	19	18	14	23	47	58
ENSMUSG(	3110001I2:	0.001614	44	55	62	84	110	103
ENSMUSG(	Gm12247	1.84E-06	1	2	3	22	70	15
ENSMUSG(	Prdx2-ps1	0.029173	3	6	9	7	27	34
ENSMUSG(	Gm13410	1.26E-09	38	32	27	118	145	82

ENSMUSG(	Rpl3-ps2	0.004073	57	54	72	370	68	227
ENSMUSG(	Gm11814	4.49E-29	5	8	4	70	92	75
ENSMUSG(	Rpl3-ps1	0.002807	500	439	510	2099	538	1278
ENSMUSG(	4930570G1	3.41E-05	65	57	66	215	223	99
ENSMUSG(	Gm12758	4.88E-22	82	63	86	10	13	5
ENSMUSG(	Gm14508	0.007178	243	321	289	157	212	151
ENSMUSG(	A930031H1	0.047729	56	57	28	109	94	65
ENSMUSG(	5330413P1	0.003575	373	325	276	178	217	212
ENSMUSG(	A230056P1	5.62E-09	95	99	117	17	41	38
ENSMUSG(	A330041J2	0.044401	8	1	5	25	14	8
ENSMUSG(	Gm16262	1.78E-05	151	181	158	307	279	270
ENSMUSG(	H2af-ps2	0.042352	1	1	1	67	3	2
ENSMUSG(	Gm6548	8.81E-06	4	11	1	22	29	24
ENSMUSG(	Gm17709	0.030388	33	34	39	110	48	62
ENSMUSG(	AA465934	0.004748	27	14	18	23	155	92
ENSMUSG(	Gm8355	3.18E-06	397	375	301	963	639	680
ENSMUSG(	Gm16867	9.39E-05	99	90	75	43	28	48
ENSMUSG(	Gm5900	8.94E-06	9	13	7	80	55	24
ENSMUSG(	2610528A1	4.33E-05	75	78	102	21	29	47
ENSMUSG(	A930038B1	6.91E-09	14	7	19	41	62	67
ENSMUSG(	Gm26670	0.006338	100	100	101	121	357	245
ENSMUSG(	Gm18190	0.002181	27	25	18	12	7	2
ENSMUSG(	Gm27177	0.000331	82	74	63	36	24	42
ENSMUSG(	Tcf24	0.001127	111	151	148	198	256	285
ENSMUSG(	B130024G1	0.000839	47	66	51	25	26	24
ENSMUSG(	Gm15459	1.08E-15	1616	1595	1193	6644	4286	4041
ENSMUSG(	Gm5920	0.048356	3	4	11	14	20	15
ENSMUSG(	4930429P2	0.001752	40	41	45	14	22	20
ENSMUSG(	4632432E1	0.021297	96	74	85	57	44	53
ENSMUSG(	Gm32460	0.001836	0	5	2	4	60	47
ENSMUSG(	Gm43622	0.000288	111	89	91	196	251	147
ENSMUSG(	Gm9260	9.94E-06	2	5	4	25	15	24
ENSMUSG(	Gm7993	1.89E-12	69	75	76	593	241	334
ENSMUSG(	Gm3375	0.020623	74	82	87	321	73	301
ENSMUSG(	Gm18609	0.017228	45	53	44	15	28	29
ENSMUSG(	Gm6210	0.005538	116	88	85	188	153	145
ENSMUSG(	Gm6916	5.55E-19	28	29	31	182	118	131
ENSMUSG(	Gm35082	4.42E-29	1	1	2	83	48	74
ENSMUSG(	Gm44812	0.046071	37	35	49	25	15	24
ENSMUSG(	Gm44954	1.24E-08	12	12	14	41	46	75
ENSMUSG(	Gm44541	0.005181	12	8	5	30	20	22
ENSMUSG(	Gm45463	4.53E-05	4	0	5	6	185	280
ENSMUSG(	Gm18860	1.70E-06	6	5	5	22	37	24
ENSMUSG(	Iqcn	0.000134	20	11	24	5	2	3
ENSMUSG(	Gm20139	0.045722	918	871	948	488	759	612
ENSMUSG(	Gm47252	8.66E-09	35	43	43	113	155	282
ENSMUSG(	Gm4065	0.044401	156	119	163	88	112	78

ENSMUSG(	Gm35206	0.003405	28	38	48	13	16	19
ENSMUSG(	Srp54b	0.028851	2199	2507	1997	1529	1795	1374
ENSMUSG(	Gm49331	0.007046	6	6	5	14	14	27
ENSMUSG(	Gm48904	0.002836	9	11	23	62	25	49
ENSMUSG(	A330084C1	0.036404	9	16	20	41	26	30
ENSMUSG(	Gm49156	0.000865	246	232	237	104	171	96
ENSMUSG(	Gm50221	4.20E-31	7	7	8	128	281	166
ENSMUSG(	Gm5503	0.021293	11	10	13	41	14	37
ENSMUSG(	2900057B2	0.001614	60	33	21	10	7	15
ENSMUSG(	Gm41793	3.57E-05	68	81	95	205	295	140
ENSMUSG(	ENSMUSG(	0.028357	34	43	30	11	24	18

Table S3. qRT-PCR primers for mouse *Abca4, Pkm1, Pkm2*, and *Rho* genes

Gene	**Forward**	**Reverse**
Abca4	AGG GAG AGC TGT GGT TCT CA	AGC AAG TCG TCC TTT GGA GA
Pkm1	CAGCCAAAGGGGACTATCCT	GAGGCTCGCACAAGTTCTTC
Pkm2	CTATCCTCTGGAGGCTGTGC	GTGGGGTCGCTGGTAATG
Rho	CAAGAATCCACTGGGAGATGA	GTGTGTGGGGACAGGAGAACT
Rpl38	CGCCATGCCTCGGAAA	CCGCCGGGCTGTCAG
Actb	ACTGGGACGACATGGAGAAG	GGGGTGTTGAAGGTCTCAAA
Rpl38, ribosomal protein L38*; Actb,* actin		

Table S4. Antibodies used for immunofluorescence (IF) and immunoblot Analysis

**Antibody raised against**	**Host species**	**Dilution**	**Manufacturer**	**Catalog number**
LDHA	Rabbit	1:1000<br>1:50 (IF)	Proteintech	21799-1-AP
LDHB	Rabbit	1:1000<br>1:50 (IF)	Proteintech	14824-1-AP
Pde6β	Mouse	1:1000<br>1:25 (IF)	Santa Cruz	SC-377486
Rhodopsin	Mouse	1:1000<br>1:50 (IF)	In-house	Gift from Dr. Jim McGinnis (OUHSC)
Rod-Arrestin	Mouse	1:1000<br>1:500 (IF)	In-house	Gift from Dr. Paul Hargrave (University of Florida)
M-opsin	Rabbit	1:1000<br>1:100 (IF)	Millipore Sigma	AB5405
S-opsin	Rabbit	1: 100 (IF)	Millipore Sigma	ABN1660-1
Cone-Arrestin	Rabbit	1:1000	Millipore Sigma	AB15282
Actin	Mouse	1:1000 (IB)	Thermo Fisher Scientific	MA1-744
Glutamine synthetase (GS)	Mouse	1:1000<br>1:50 (IF)	Abcam	Ab64613
Glial fibrillary acid protein (GFAP)	Rabbit	1:100 (IF)	Dako	20334
PKM1	Rabbit mAb	1:1000<br>1:50 (IF)	Cell Signaling Technology	7067
PKM2	Rabbit mAb	1:1000<br>1:100 (IF)	Cell Signaling Technology	4053
Phospho-PKM2 (Tyr105)	Rabbit	1:1000<br>1:50 (IF)	Cell Signaling Technology	3827
Aldolase C	Mouse	1:1000<br>1:100 (IF)	EnCor Biotechnology, Inc.	MCA-4A9
Glut1	Rabbit	1:1000<br>1:25 (IF)	Novus Biologicals	NB110-39113
RBPMS	Rabbit	1:1000	GeneTex	GTX118619
Abca4	Mouse	1:1000	Millipore Sigma	MABN1775
RPE65	Rabbit	1:1000	In-house	Gift from Dr. Jian-Xing Ma (University of Oklahoma)
Occludin	Rabbit	1:1000	Proteintech	27260-1-AP
ZO1-Alexa Fluor™ 594	Mouse	1:50 (IF)	ThermoFisher Scientific	339194
